# Comparison of the antiremodeling effects of losartan and mirabegron in a rat model of uremic cardiomyopathy

**DOI:** 10.1038/s41598-021-96815-5

**Published:** 2021-09-01

**Authors:** Zsuzsanna Z. A. Kovács, Gergő Szűcs, Marah Freiwan, Mónika G. Kovács, Fanni M. Márványkövi, Hoa Dinh, Andrea Siska, Katalin Farkas, Ferenc Kovács, András Kriston, Péter Horváth, Bence Kővári, Bálint Gábor Cserni, Gábor Cserni, Imre Földesi, Tamás Csont, Márta Sárközy

**Affiliations:** 1 MEDICS Research Group, Department of Biochemistry, Interdisciplinary Center of Excellence, Albert Szent-Györgyi Medical School, University of Szeged, Dóm tér 9, Szeged, 6720 Hungary; 2 Department of Laboratory Medicine, Albert Szent-Györgyi Medical School, University of Szeged, Semmelweis utca 6, Szeged, 6720 Hungary; 3 Synthetic and Systems Biology Unit, Biological Research Centre, Eötvös Loránd Research Network, Temesvári krt. 62, Szeged, 6726 Hungary; 4 Single-Cell Technologies Ltd, Temesvári krt. 62, Szeged, 6726 Hungary; 5 Institute for Molecular Medicine Finland (FIMM), University of Helsinki, 00014 Helsinki, Finland; 6 Department of Pathology, Albert Szent-Györgyi Medical School, University of Szeged, Állomás utca 1, Szeged, 6720 Hungary

**Keywords:** Heart failure, Chronic kidney disease, Preclinical research

## Abstract

Uremic cardiomyopathy is characterized by diastolic dysfunction (DD), left ventricular hypertrophy (LVH), and fibrosis. Angiotensin-II plays a major role in the development of uremic cardiomyopathy via nitro-oxidative and inflammatory mechanisms. In heart failure, the beta-3 adrenergic receptor (β3-AR) is up-regulated and coupled to endothelial nitric oxide synthase (eNOS)-mediated pathways, exerting antiremodeling effects. We aimed to compare the antiremodeling effects of the angiotensin-II receptor blocker losartan and the β3-AR agonist mirabegron in uremic cardiomyopathy. Chronic kidney disease (CKD) was induced by 5/6th nephrectomy in male Wistar rats. Five weeks later, rats were randomized into four groups: (1) sham-operated, (2) CKD, (3) losartan-treated (10 mg/kg/day) CKD, and (4) mirabegron-treated (10 mg/kg/day) CKD groups. At week 13, echocardiographic, histologic, laboratory, qRT-PCR, and Western blot measurements proved the development of uremic cardiomyopathy with DD, LVH, fibrosis, inflammation, and reduced eNOS levels, which were significantly ameliorated by losartan. However, mirabegron showed a tendency to decrease DD and fibrosis; but eNOS expression remained reduced. In uremic cardiomyopathy, β3-AR, sarcoplasmic reticulum ATPase (SERCA), and phospholamban levels did not change irrespective of treatments. Mirabegron reduced the angiotensin-II receptor 1 expression in uremic cardiomyopathy that might explain its mild antiremodeling effects despite the unchanged expression of the β3-AR.

## Introduction

Chronic kidney disease (CKD) is a global health problem affecting 1 out of 10 people due to the growing prevalence of its primary causes, including aging, diabetes mellitus, and hypertension^1,2^. CKD patients have a five- to tenfold higher risk for developing cardiovascular diseases compared to the age-matched non-CKD population^3^. The CKD-associated chronic structural, functional, and electrophysiological remodeling of the heart is called uremic cardiomyopathy (i.e., type 4 cardiorenal syndrome)^4,5^. It is characterized by diastolic dysfunction (DD), left ventricular hypertrophy (LVH), capillary rarefaction, endothelial dysfunction, and fibrosis in the stage of heart failure with preserved ejection fraction (HFpEF)^5,6^. With the progression of CKD, cardiac fibrosis becomes more prominent, leading to systolic dysfunction in the phase of heart failure with reduced ejection fraction (HFrEF)^5,6^. Moreover, uremic cardiomyopathy markedly enhances the susceptibility of the heart to further injuries, including acute myocardial infarction and arrhythmias^5–7^. Therefore, cardiovascular diseases are more commonly fatal in CKD patients than in the general population without CKD^8^.

Multiple factors might contribute to the development of uremic cardiomyopathy; however, the precise molecular mechanisms are not entirely clear. These factors include non-CKD-specific mechanisms such as hemodynamic overload with over-activation of the renin–angiotensin–aldosterone system (RAAS) and sympathetic nervous system, hypertension, endothelial dysfunction, inflammation, and increased nitro-oxidative stress and CKD-specific factors such as circulating uremic toxins and renal anemia^5,6^. In uremic cardiomyopathy, the chronically elevated angiotensin-II levels might stimulate several pathological mechanisms such as inflammation, nitro-oxidative stress, decreased NO bioavailability via angiotensin-II type 1 receptors (AT1), ultimately leading to fibrosis both in the heart and kidneys^9–12^. Therefore, RAAS inhibitors, including angiotensin-II receptor blockers (ARBs), are widely used to slow down heart failure and CKD progression in clinical practice^12,13^. Despite the broad availability of standard heart failure medications such as ARBs, cardiovascular morbidity and mortality among CKD patients remained high^5^. Therefore, using novel agents that ameliorate or prevent the progression of uremic cardiomyopathy is urgently needed.

Mirabegron is a β3-AR agonist recently used in the clinical treatment of overactive bladder syndrome^14^. In healthy heart tissue, the expression of β3-AR is considered low in atrial and ventricular myocytes but more abundant in non-cardiomyocytes, including endothelial cells^14–16^. In contrast to β1- and β2-ARs, cardiac β3-AR abundance increases as a counterregulatory mechanism to prevent chronic adrenergic overactivation in chronic ischemia and heart failure^14,15,17–19^. In pre-clinical models, the β3-AR agonists attenuated cardiac hypertrophy and fibrosis and improved cardiac contractility via coupling of β3-AR to the eNOS/cGMP pathway and activating Na^+^/K^+^-ATPase-mediated Na^+^ export in the cardiomyocytes. Moreover, the antioxidant effects of the β3-AR signaling may protect the heart from elevated nitro-oxidative stress and the consecutive pro-inflammatory processes^14,20,21^. Belge et al*.* demonstrated that angiotensin-II administration did not induce cardiac fibrosis and hypertrophy in mice with cardiomyocyte-specific expression of human β3-AR^22^. It has also been described in pancreatic^23^ and lung tissues^24^ of male apoE knock-out mice that chronic administration of the β3-AR agonist BRL37344 could down-regulate the AT1 receptors. These results suggest that chronic β3-adrenoceptor activation can regulate the expression of angiotensin-II receptors, and these interactions may play a protective role in the pancreas and lungs or other tissues such as the heart.

Therefore, the antiremodeling effects of the β3-AR agonist mirabegron are investigated in HFpEF and HFrEF patients in clinical trials. However, late-stage CKD patients are routinely excluded from clinical trials. Thus, the effects of mirabegron were not investigated in a selected patient population with mild to moderate uremic cardiomyopathy or in experimental CKD. Therefore, our present study aimed to compare the antiremodeling effects of the ARB losartan used in standard heart failure therapy and the novel β3-AR agonist mirabegron in uremic cardiomyopathy in rats.

## Results

### Neither losartan nor mirabegron improved the severity of CKD based on serum and urine parameters

**Figure 1:**
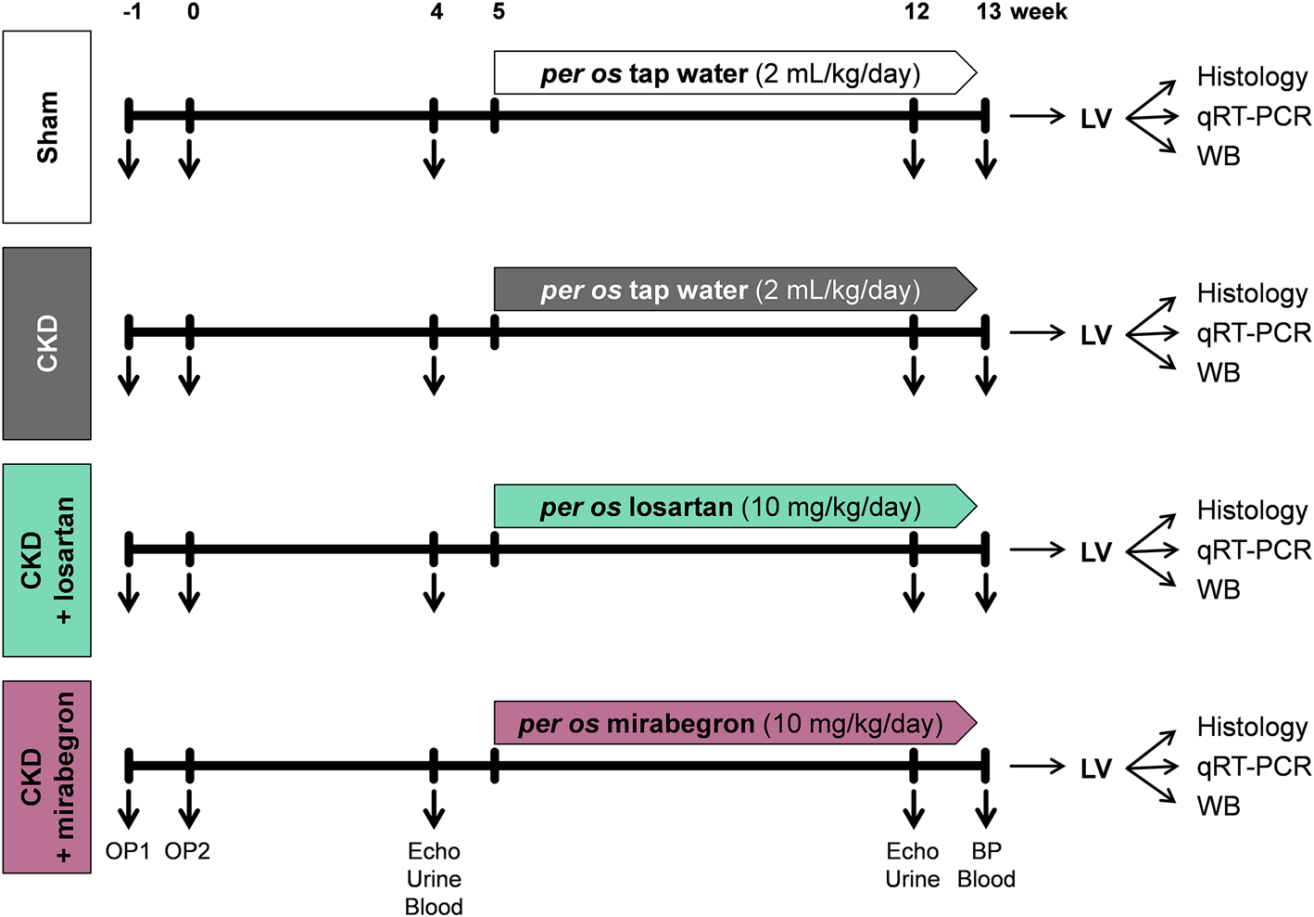
Experimental setup. *BP* blood pressure, *CKD* chronic kidney disease, *LV* left ventricle, *OP* operation, *WB* Western blot.

**Figure 2:**
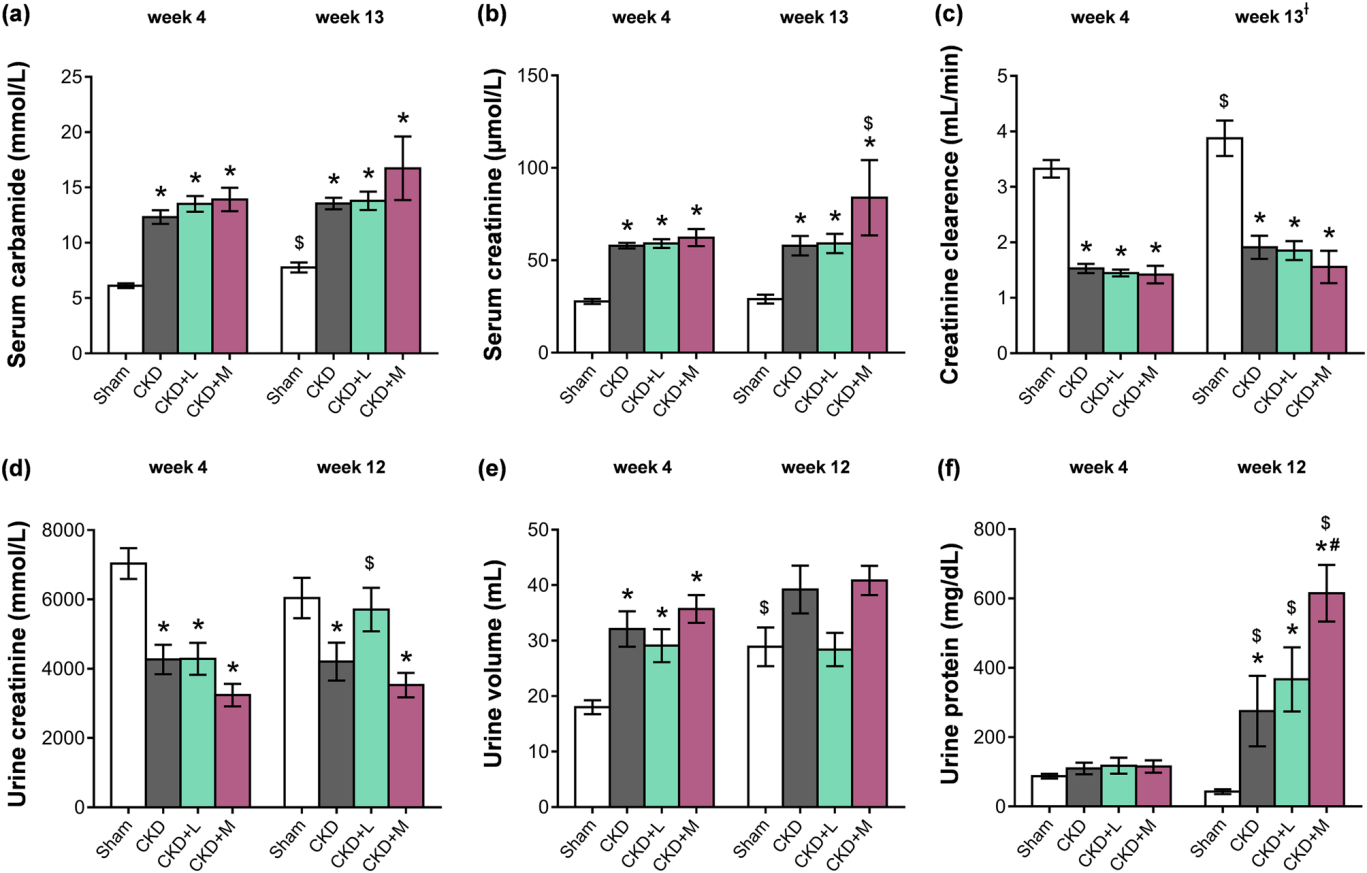
The effects of losartan and mirabegron on the development of CKD in 5/6th nephrectomized rats. (**a**) Serum carbamide concentration, (**b**) serum creatinine concentration, (**c**) creatinine clearance, (**d**) urine creatinine concentration, (**e**) urine volume, and (**f**) urine protein concentration at week 4 and the endpoint. Values are presented as mean ± S.E.M., *p < 0.05 vs. sham-operated group and ^#^p < 0.05 vs. CKD group (n = 7–10, One-Way ANOVA, Holm-Sidak post hoc test), ^$^p < 0.05 vs. week 4 in the same group (n = 7–10, Two-ways repeated-measures ANOVA, Holm-Sidak post hoc test). *Sham* sham-operated group, *CKD* chronic kidney disease group, *CKD + L* losartan-treated chronic kidney disease group, *CKD + M* mirabegron-treated chronic kidney disease group. Creatinine clearance was calculated according to the standard formula (urine creatinine concentration [μM] × urine volume for 24 h [mL])/(serum creatinine concentration [μM] × 24 × 60 min). ^†^At the endpoint, urine volume and creatinine concentration were measured at week 12 and serum creatinine concentration at week 13.

At the 4th and 12th or 13th follow-up weeks, serum and urine metabolite concentrations were determined to verify the development of CKD induced by 5/6th nephrectomy (Fig. 1). Serum carbamide and creatinine levels were significantly increased in the 5/6th nephrectomized groups irrespective of losartan or mirabegron treatment compared to the sham-operated group at weeks 4 and 13, respectively (Fig. 2a,b). In the mirabegron-treated CKD group, serum creatinine concentration was higher at the endpoint compared to the week 4 values, suggesting a worsening renal function (Fig. 2b). Creatinine clearance decreased significantly in the 5/6th nephrectomized groups irrespective of losartan or mirabegron treatment at weeks 4 and 13 compared to the sham-operated rats at the corresponding follow-up timepoint, implicating an impaired renal function (Fig. 2c). At week 4, urine creatinine concentration was significantly decreased in the 5/6th nephrectomized groups irrespective of treatments compared to the sham-operated group (Fig. 2d). At week 12, urine creatinine concentration was significantly lower in the CKD and mirabegron-treated CKD groups as compared to the sham-operated group (Fig. 2d). In response to losartan, the urine creatinine concentration was not different from the sham-operated group and showed a tendency of increase as compared to the CKD group (p = 0.109) (Fig. 2d). In the losartan-treated CKD group, urine creatinine concentration was significantly higher at the endpoint compared to the week 4 value, indicating improved creatinine excretion into the urine in response to losartan (Fig. 2d). At week 4, urine volumes were significantly higher in the 5/6th nephrectomized groups irrespective of treatments indicating the development of the polyuric phase of CKD (Fig. 2e). At week 12, urine volume showed a tendency of increase in the CKD group compared to the sham-operated group (p = 0.082) (Fig. 2e). However, it was not significantly different between the sham-operated and the losartan-treated groups and showed a tendency of decrease in response to losartan as compared to the CKD group (p = 0.086) (Fig. 2e). In contrast, urine volume was not different between the CKD and mirabegron-treated CKD groups at week 12 (Fig. 2e). At week 4, there was no difference in the urine protein concentrations between the groups (Fig. 2f). At week 12, urine protein concentration was significantly higher in the CKD groups as compared to the sham-operated group showing an impaired glomerular function (Fig. 2f). At week 12, losartan did not affect, but mirabegron significantly increased the proteinuria compared to the CKD group, further worsening the glomerular function in CKD (Fig. 2f). Urine protein concentrations were significantly higher at week 12 than the week 4 values in the CKD groups, irrespective of losartan or mirabegron treatments indicating a worsening glomerular function in CKD (Fig. 2f). In the sham-operated group, serum carbamide concentration, creatinine clearance, and urine volume were higher at the endpoint as compared to the week 4 values, probably due to the growth of the animals (Fig. 2a,c,e). In the CKD groups, irrespective of treatments, these parameters did not increase significantly at the endpoint compared to the week 4 values.

To further characterize the severity of CKD, serum ion levels were determined at week 13 (Table 1). There was no significant difference in the serum sodium, potassium, chloride, and phosphate ion levels between the groups (Table 1). Serum calcium ion concentrations were significantly increased in the CKD groups irrespective of losartan- or mirabegron-treatment compared to the sham-operated group (Table 1). Serum magnesium ion concentrations were not significantly different between the CKD and the sham-operated groups (Table 1). Losartan did not change the serum magnesium ion concentration significantly compared to the CKD group (Table 1). However, serum magnesium ion concentration was significantly elevated in the mirabegron-treated CKD group as compared to the sham-operated or CKD groups (Table 1).

**Table 1:** Serum and total blood count parameters at week 13.

Parameter (unit)	Groups			
	Sham	CKD	CKD + losartan	CKD + mirabegron
Serum sodium ion (mmol/L)	141 ± 0.7	140 ± 0.43	141 ± 0.62	141 ± 0.74
Serum potassium ion (mmol/L)	5.3 ± 0.16	4.97 ± 0.17	5.53 ± 0.15	5.23 ± 0.31
Serum chloride ion (mmol/L)	103 ± 0.95	103 ± 0.51	102 ± 0.45	101 ± 1.07
Serum phosphate ion (mmol/L)	2.28 ± 0.15	2.31 ± 0.1	2.22 ± 0.06	2.46 ± 0.13
Serum calcium ion (mmol/L)	2.44 ± 0.01	2.54 ± 0.04*	2.58 ± 0.02*	2.59 ± 0.02*
Serum magnesium ion (mmol/L)	0.9 ± 0.02	0.97 ± 0.04	1.03 ± 0.04	1.29 ± 0.12*^#^
Serum total cholesterol (mmol/L)	1.56 ± 0.03	2.23 ± 0.2*	2.65 ± 0.12*	4.11 ± 0.22*^#^
Serum LDL-cholesterol (mmol/L)	0.53 ± 0.04	0.79 ± 0.08*	0.73 ± 0.03*	1.16 ± 0.11*^#^
Serum triglyceride (mmol/L)	0.52 ± 0.03	0.6 ± 0.07	0.77 ± 0.12	0.99 ± 0.1*^#^
Red blood cells (10^12^/L)	8.3 ± 0.11	7.25 ± 0.23*	7.09 ± 0.22*	6.55 ± 0.32*
Hematocrit (%)	44 ± 1	39 ± 1*	39 ± 1*	36 ± 1*^#^
Hemoglobin (g/L)	148 ± 2.29	131 ± 4.2*	131 ± 2.26*	120.14 ± 4.58*
White blood cells (10^9^/L)	3.45 ± 0.23	6.63 ± 0.87*	4.47 ± 0.45^#^	6.57 ± 0.6*

Values are presented as mean ± S.E.M., *p < 0.05 vs. sham-operated group, ^#^p < 0.05 vs. CKD group (n = 6–10 for serum parameters, n = 5–8 for blood count parameters, One-Way ANOVA, Holm-Sidak post hoc test).

*Sham* sham-operated group, *CKD* chronic kidney disease group.

### Mirabegron increased the serum cholesterol level in CKD

Serum total cholesterol, low-density lipoprotein (LDL) cholesterol, and triglyceride levels were measured as cardiovascular risk factors at week 13 (Table 1). Serum total cholesterol and LDL-cholesterol levels elevated significantly, and serum triglyceride levels did not change significantly in the CKD group compared to the sham-operated group (Table 1). Both in the losartan- and mirabegron-treated CKD groups, total serum cholesterol, and LDL-cholesterol levels were significantly elevated compared to the sham-operated group; however, mirabegron further elevated the total cholesterol and LDL-cholesterol levels compared to the CKD group (Table 1). Moreover, serum triglyceride level was significantly higher in the mirabegron-treated CKD group as compared to sham-operated or CKD groups (Table 1).

### Losartan reduced the white blood cell count, but mirabegron worsened the renal anemia

Blood count parameters were determined at week 13 to characterize the severity of the systemic inflammation and anemia associated with CKD (Table 1). In the CKD groups, irrespective of losartan- or mirabegron-treatment, the red blood cell count, hematocrit, and hemoglobin levels were significantly decreased compared to the sham-operated group, indicating the development of renal anemia at week 13 (Table 1). Moreover, the hematocrit level was significantly lower in the mirabegron-treated CKD group compared to the CKD group. The white blood cell count was significantly higher in the CKD group than the sham-operated group pointing out the development of systemic inflammation (Table 1). However, losartan significantly reduced the white blood cell count as compared to the CKD group (Table 1). Mirabegron did not change the white blood cell count in CKD (Table 1).

### The systolic function remained unchanged in uremic cardiomyopathy irrespective of losartan or mirabegron-treatment

**Figure 3:**
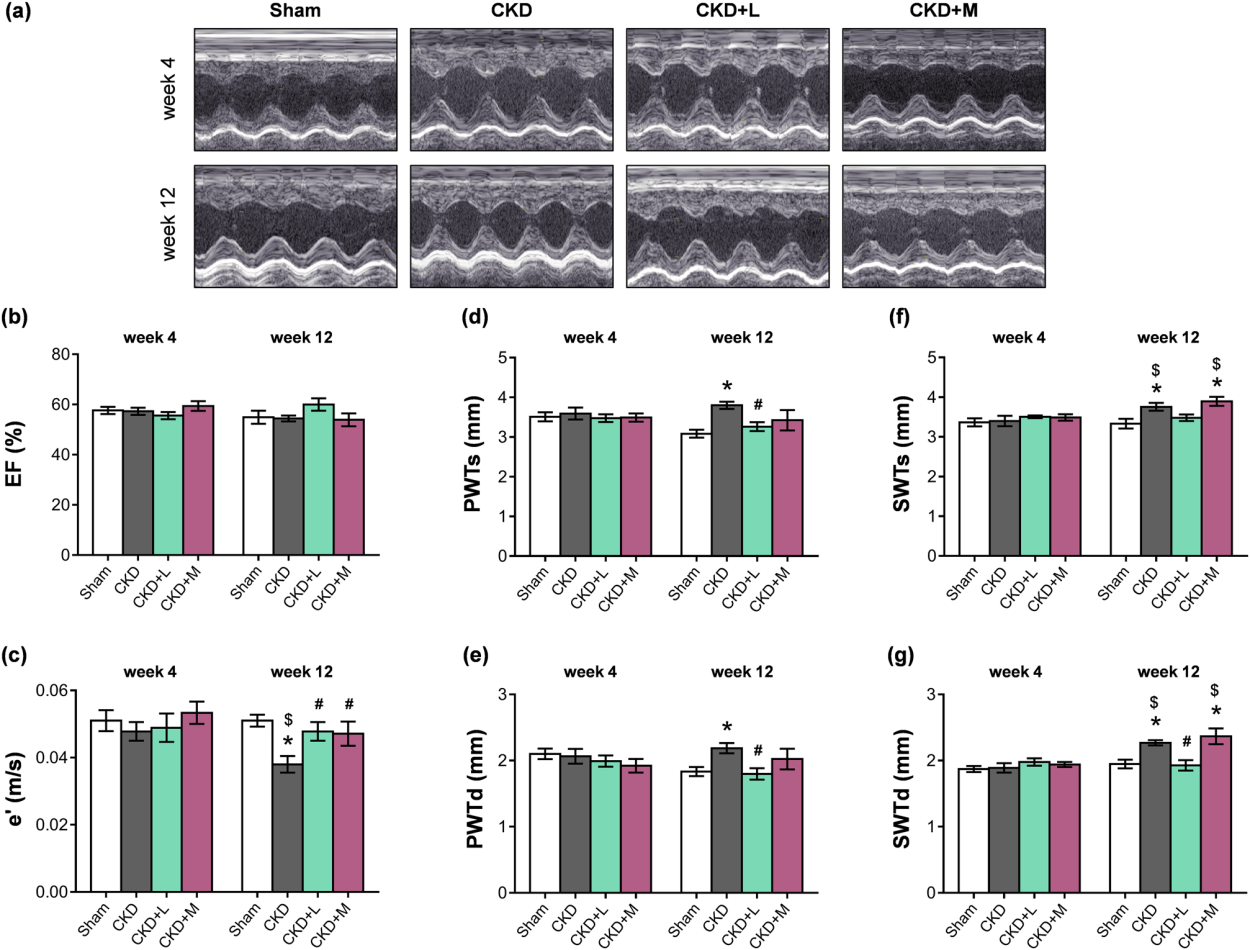
The effects of losartan and mirabegron on the echocardiographic parameters at week 13. (**a**) Representative M-mode images, (**b**) ejection fraction (EF), (**c**) diastolic septal mitral annulus velocity (e'), (**d**) posterior wall thicknesses in systole (PWTs) and (**e**) diastole (PWTd), (**f**) septal wall thickness in systole (SWTs) and (**g**) diastole (SWTd). Values are presented as mean ± S.E.M., *p < 0.05 vs. sham-operated group, ^#^p < 0.05 vs. CKD group (n = 7–10, One-Way ANOVA, Holm-Sidak post hoc test), ^$^p < 0.05 vs. week 4 in the same group (n = 7–10, Two-ways repeated-measures ANOVA, Holm-Sidak post hoc test). *Sham* sham-operated group, *CKD* chronic kidney disease group, *CKD + L* losartan-treated chronic kidney disease group, *CKD + M* mirabegron-treated chronic kidney disease group. Representative M-mode images were saved from the EchoPac Dimension v201 software.

Transthoracic echocardiography was performed at week 4 prior to the treatments and at week 12 to monitor the effects of CKD and the medications on cardiac morphology and function (Figs. 1 and 3a–g). The main systolic parameter, the ejection fraction (EF), was not significantly different in the sham-operated and CKD groups, irrespective of treatments, at weeks 4 and 12 or in the same group between weeks 4 and 12 (Fig. 3b). The heart rate (HR) was not significantly different between the CKD and sham-operated groups (Table 2). HR did not change significantly in response to losartan in CKD; however, it was significantly reduced by mirabegron compared to the CKD group (Table 2). The left ventricular end-systolic volume (LVESV), left ventricular end-diastolic volume (LVEDV), and stroke volume (SV) were similar in the CKD and sham-operated groups (Table 2). Losartan did not change the LVESV, LVEDV, and SV compared to the CKD group (Table 2). In contrast, the mirabegron-treated CKD group showed significantly higher LVESV and LVEDV compared to the sham-operated or CKD groups (Table 2). The SV was also significantly elevated in the mirabegron-treated CKD group compared to the CKD group, probably due to the positive inotropic effects of mirabegron via β1-ARs^25^ (Table 2). However, the cardiac output (CO) was not different between the groups due to the compensatory effects of heart rate changes in response to the mirabegron-treatment (Table 2). The systolic parameter, isovolumic contraction time (IVCT), was significantly shorter in the CKD group as compared to the sham-operated group pointing out a mild systolic dysfunction in CKD (Table 2). IVCT did not change in response to losartan; however, it was significantly increased by mirabegron-treatment compared to the CKD group, probably due to the lower heart rate in the mirabegron-treated CKD group (Table 2).

**Table 2:** Left ventricular morphological and functional parameters assessed by transthoracic echocardiography at week 12 and blood pressure values at week 13.

Parameter (unit)	Groups			
	Sham	CKD	CKD + losartan	CKD + mirabegron
HR (1/min)	361 ± 9	386 ± 9	383 ± 13	332 ± 4^#^
LVEDV (μl)	275 ± 12	262 ± 19	262 ± 17	368 ± 33*^#^
LVESV (μl)	124 ± 8	120 ± 10	107 ± 11	181 ± 26*^#^
SV (μl)	152 ± 11	142 ± 10	161 ± 9	181 ± 8^#^
CO (mL/min)	55 ± 4	55 ± 4	61 ± 4	60 ± 3
IVCT (ms)	13.3 ± 0.67	10.8 ± 0.59*	12.22 ± 0.66	14.43 ± 0.61^#^
IVRT (ms)	14.3 ± 0.72	11.7 ± 0.47*	12.78 ± 0.43	14.71 ± 1.15^#^
E-velocity (m/s)	0.97 ± 0.03	1.02 ± 0.05	1.06 ± 0.04	1.06 ± 0.06
E/e’	19.45 ± 1.28	28.21 ± 2.66*	22.82 ± 1.55	22.88 ± 1.39
AWTs (mm)	3.19 ± 0.1	3.65 ± 0.09*	3.41 ± 0.05	3.56 ± 0.1*
AWTd (mm)	1.96 ± 0.03	2.17 ± 0.04*	1.98 ± 0.04^#^	2.24 ± 0.11*
IWTs (mm)	3.01 ± 0.11	3.69 ± 0.1*	3.32 ± 0.13	3.51 ± 0.29
IWTd (mm)	1.82 ± 0.06	2.18 ± 0.05*	1.92 ± 0.06^#^	1.99 ± 0.19
SBP (mmHg)	145 ± 4	166 ± 13	142 ± 4	170 ± 9
DBP (mmHg)	104 ± 2	122 ± 10	102 ± 3	127 ± 6
MBP (mmHg)	119 ± 3	139 ± 11	117 ± 3	144 ± 8

Values are presented as mean ± S.E.M., *p < 0.05 vs. sham-operated group and ^#^p < 0.05 vs. CKD group (n = 7–10 for echocardiography and n = 6–8 for blood pressure parameters, One-Way ANOVA, Holm-Sidak post hoc test).

*Sham* sham-operated group, *CKD* chronic kidney disease group, *AWTd* diastolic anterior wall thickness, *AWTs* systolic anterior wall thickness *CO* cardiac output, *DBP* diastolic blood pressure, *E-velocity* early ventricular filling velocity, *e’-velocity* diastolic septal mitral annulus velocity, *HR* heart rate, *IVCT* isovolumic contraction time, *IVRT* isovolumic relaxation time, *IWTd* diastolic inferior wall thickness, *IWTs* systolic inferior wall thickness, *LVEDV* left ventricular end-diastolic volume, *LVESV* left ventricular end-systolic volume, *MBP* mean arterial blood pressure, *SBP* systolic blood pressure, *SV* stroke volume.

### Both losartan and mirabegron ameliorated the diastolic dysfunction in uremic cardiomyopathy

The diastolic parameter, isovolumic relaxation time (IVRT) was significantly shorter in the CKD group as compared to the sham-operated group (Table 2). In response to losartan-treatment, IVRT was not different from the CKD or sham-operated groups (Table 2). Mirabegron-treatment significantly increased the IVRT as compared to the CKD group (Table 2). However, another diastolic parameter, the mitral valve early flow velocity (E) was not significantly different between the groups (Table 2). A sensitive parameter of the diastolic function, the septal mitral annulus velocity (e'), was significantly decreased in the CKD group in contrast to the sham-operated group indicating the development of DD in CKD (Fig. 3c). The e' velocity was significantly increased by losartan and mirabegron compared to the CKD group (Fig. 3c). E/e' ratio was significantly higher in the CKD group than the sham-operated group, which is another indicator of the DD (Table 2). In response to losartan or mirabegron, the E/e' ratio was not significantly different from the sham-operated group (Table 2). In the sham-operated group, e' was not significantly different at weeks 4 and 12 (Fig. 3c). In the CKD group, e' was significantly decreased at week 12 as compared to the week 4 value, verifying the progression of diastolic dysfunction in uremic cardiomyopathy. (Fig. 3c). Both in the losartan and mirabegron-treated groups, e' did not change at week 12 as compared to week 4 values, proving that both drugs prevented the progression of DD (Fig. 3c).

### Losartan but not mirabegron ameliorated the echocardiographic signs of left ventricular hypertrophy in CKD

Four weeks after the operations, there was no significant difference in any cardiac morphologic parameters between the groups (Fig. 3d–g and Supplementary Table S1). At week 12, systolic and diastolic anterior, inferior, posterior, and septal wall thicknesses were significantly increased in response to CKD, indicating the development of a concentric LVH (Fig. 3d–g and Table 2). Losartan significantly reduced the diastolic anterior, inferior, posterior, and septal, as well as the systolic posterior wall thicknesses compared to the CKD group (Fig. 3d,e,g, and Table 2). Losartan did not change the systolic anterior, inferior, and septal wall thicknesses significantly (Fig. 3f and Table 2). In contrast, mirabegron failed to significantly reduce the wall thicknesses in uremic cardiomyopathy (Fig. 3d–g and Table 2). There was no significant difference in the sham-operated group at week 4 and 12 values in cardiac morphology (Fig. 3d–g). In the CKD group, the systolic and diastolic septal wall thicknesses were significantly increased at week 12 as compared to the week 4 values, verifying the progression of LVH (Fig. 3f,g). In the losartan-treated CKD group, systolic and diastolic septal wall thicknesses did not increase at week 12 compared to the week 4 values, pointing out that losartan prevented the development of LVH (Fig. 3f,g). In contrast, in the mirabegron-treated group, systolic and diastolic wall thicknesses were significantly higher at week 12 than the week 4 values, proving that mirabegron failed to prevent the progression of LVH (Fig. 3f,g). In CKD, hypertension is a well-known complication and an independent risk factor for the development of LVH. In our study, the systolic, diastolic, and mean arterial blood pressure values were not statistically different between the groups at week 13 (Table 2).

### Losartan but not mirabegron reduced the left ventricular weight in uremic cardiomyopathy

At week 13, hearts, lungs, left kidneys, and tibias were isolated, then left and right ventricles were separated, and the organ weights and tibia lengths were measured (Supplementary Table S2). There was no significant difference in body weight, tibia length, right ventricular weight between the groups (Supplementary Table S2). However, LV weight showed a significant increase in the CKD group compared to the sham-operated group indicating the macroscopic signs of LVH (Supplementary Table S2). Losartan but not mirabegron significantly decreased the heart weight and LV weight compared to the CKD group supporting the echocardiographic results (Supplementary Table S2, Table 2, and Fig. 3). The remnant left kidney weight was markedly higher in the CKD group than the whole left kidney weight in the sham-operated group, pointing out a frank compensatory renal hypertrophy in CKD (Table S2). In response to losartan, the remnant left kidney weight was not different from the sham-operated or the CKD groups (Supplementary Table S2). Mirabegron did not reduce the remnant left kidney weight in CKD (Supplementary Table S2). Lung weight was not significantly different between the CKD and the sham-operated groups (Supplementary Table S2). In response to losartan, lung weight was not different from the sham-operated and CKD groups (Supplementary Table S2). In contrast, the mirabegron-treated CKD group showed significantly higher lung weights as compared to the sham-operated group suggesting the presence of pulmonary edema (Supplementary Table S2).

### Losartan but not mirabegron attenuated cardiomyocyte hypertrophy in CKD

**Figure 4:**
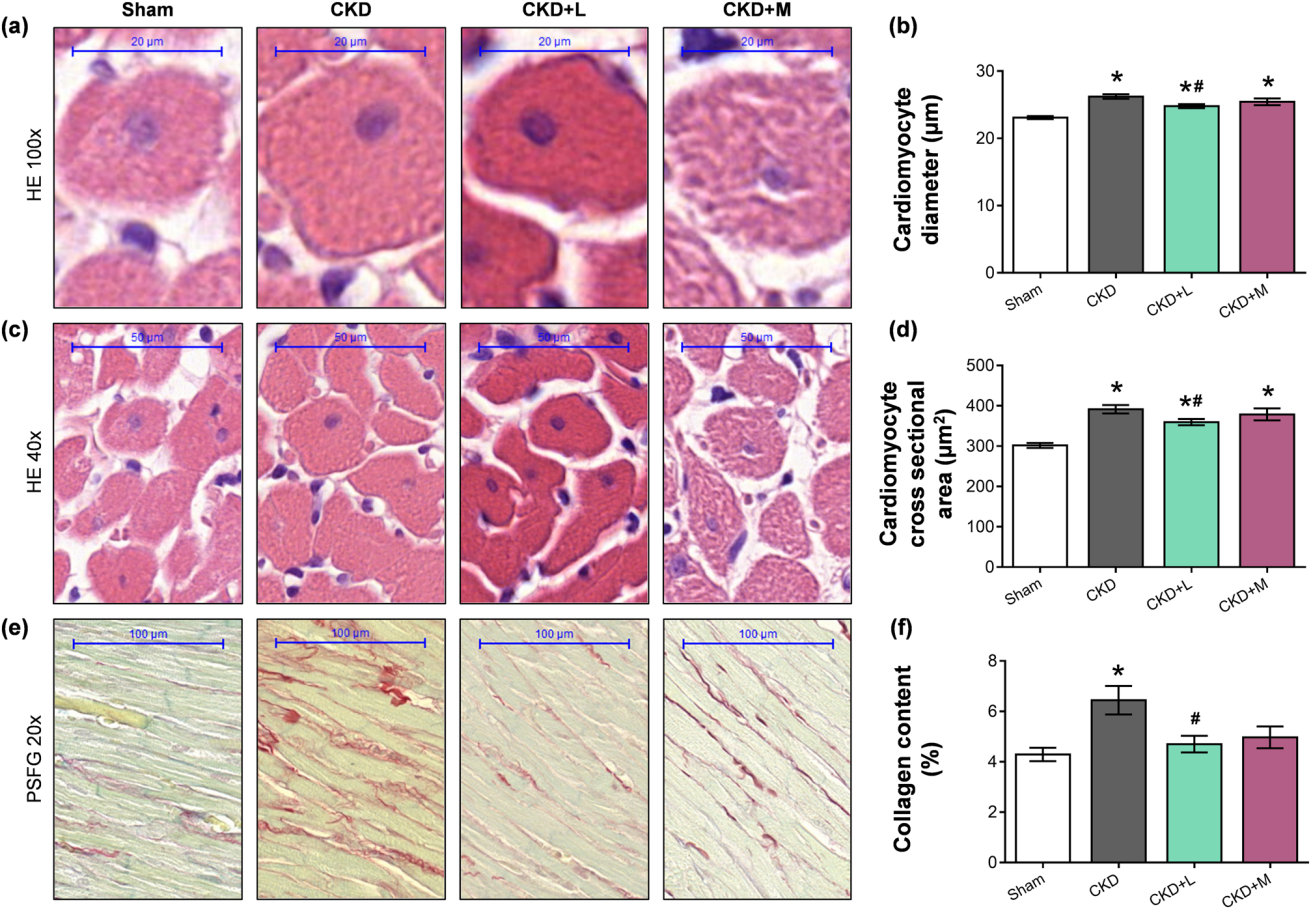
The effects of losartan and mirabegron on left ventricular hypertrophy and fibrosis assessed by histology at week 13. (**a**) Representative haematoxylin–eosin (HE)-stained slides at 100 × and (**c**) 40 × magnification, (**b**) cardiomyocyte diameters and (**d**) cross-sectional areas, (**e**) representative picrosirius red and fast green (PSFG)-stained slides at 20 × magnification, (**f**) left ventricular collagen content. On the digital HE images, cardiomyocyte diameters and cross-sectional areas were measured in 100 selected cardiomyocytes on left ventricular sections cut on the same plane. The mean values of the collagen content of 10 representative PSFG-stained images were calculated and used for statistical evaluation in the case of each left ventricular slide. Scale bars represent 20 µm at the 100 × magnified images, 50 µm at the 40 × magnified images, and 100 µm at the 20 × magnified images. Values are presented as mean ± S.E.M., *p < 0.05 vs. sham-operated group, ^#^p < 0.05 vs. CKD group. (n = 7–10, One-Way ANOVA, Holm-Sidak post hoc test). *Sham* sham-operated group, *CKD* chronic kidney disease group, *CKD + L* losartan-treated chronic kidney disease group, *CKD + M* mirabegron-treated chronic kidney disease group. Representative HE- or PSFG-stained slides were captured in the Panoramic Viewer 1.15.4 software.

Cardiomyocyte diameters and cross-sectional areas were measured on hematoxylin–eosin-stained histological slides to verify the development of LVH assessed by echocardiography and autopsy. Cardiomyocytes showed a significantly enlarged diameter and cross-sectional area in the CKD group compared to the sham-operated group, confirming the development of LVH at the cellular level (Fig. 4a–d). Losartan but not mirabegron treatment significantly reduced the cardiomyocyte diameter and cross-sectional area in CKD, validating the echocardiographic and macroscopic findings (Fig. 4a–d). To strengthen our results at the molecular level, the expression of cardiac hypertrophy markers was measured by qRT-PCR. The increased ratio of the fetal myosin heavy chain β-isoform to the adult α-isoform (β-MHC to α-MHC ratio) is an indicator of the fetal gene reprogramming in LVH in response to tissue hypoxia^26^. The LV mRNA expression of α-MHC (*Myh6*) and β-MHC (*Myh7*) isoforms, as well as their ratio, failed to change significantly in the CKD group as compared to the sham-operated group, suggesting a slowdown phase in the LVH development at week 13 (Table 3). Losartan but not mirabegron significantly reduced the LV expression of the β-MHC isoform (*Myh7*) and the β-MHC to α-MHC ratio compared to the CKD group (Table 3). In contrast, mirabegron significantly reduced the α-MHC isoform (*Myh6*) compared to the CKD or sham-operated groups (Table 3), probably, due to its negative inotropic effects^27^.

**Table 3:** qRT-PCR results at week 13.

Gene/house-keeping gene	Groups			
	Sham	CKD	CKD + losartan	CKD + mirabegron
*Myh6/Ppia*	1.41 ± 0.07	1.23 ± 0.11	1.21 ± 0.07	0.76 ± 0.09*^#^
*Myh7/Ppia*	1.29 ± 0.1	1.27 ± 0.18	0.71 ± 0.12*^#^	1.01 ± 0.14
*Myh6/Myh7*	0.94 ± 0.09	1.26 ± 0.15	0.61 ± 0.13^#^	1.32 ± 0.31
*Col1a1/Ppia*	0.2 ± 0.05	0.32 ± 0.08	0.25 ± 0.04	0.19 ± 0.06
*Ctgf/Ppia*	0.93 ± 0.09	1.32 ± 0.15*	0.82 ± 0.08^#^	1.91 ± 0.24*^#^
*Nppa/Ppia*	0.42 ± 0.09	0.63 ± 0.15	0.24 ± 0.02^#^	0.56 ± 0.05
*Nppb/Ppia*	1.05 ± 0.15	1.23 ± 0.11	0.64 ± 0.09^#^	1.19 ± 0.16
*Nox4/Ppia*	1.75 ± 0.18	2.38 ± 0.20	0.57 ± 0.09*^#^	1.25 ± 0.13*^#^
*Nos2/Ppia*	0.95 ± 0.19	2.80 ± 0.55*	1.26 ± 0.21^#^	1.17 ± 0.37^#^
*IL1/Ppia*	0.77 ± 0.05	1.18 ± 0.11*	0.78 ± 0.06^#^	0.94 ± 0.16
*IL6/Ppia*	0.81 ± 0.18	2.94 ± 0.6*	0.37 ± 0.08^#^	0.52 ± 0.12^#^
*Tnf-α/Ppia*	0.77 ± 0.05	1.2 ± 0.1*	0.83 ± 0.09^#^	0.99 ± 0.12
*Agtr1a/Ppia*	1.55 ± 0.06	1.13 ± 0.1*	1.26 ± 0.11	0.86 ± 0.08*^#^
*Agt/Ppia*	1.32 ± 0.09	1.24 ± 0.09	1.08 ± 0.12	1.12 ± 0.14
*Adrb3/Ppia*	0.65 ± 0.10	0.99 ± 0.07	0.9 ± 0.1	1.08 ± 0.21

Values are presented as mean ± S.E.M., *p < 0.05 vs. sham-operated group, ^#^p < 0.05 vs. CKD group (n = 6–10, One-Way ANOVA, Holm-Sidak post hoc test).

*Sham* sham-operated group, *CKD* chronic kidney disease group, *Adrb3* β-3 adrenergic receptor*, Agt* angiotensinogen*, Agtr1a* angiotensin-II receptor type 1a*, Col1a1* collagen type 1 alpha 1 chain*, Ctgf* connective tissue growth factor*, IL1* interleukin-1*, IL6* interleukin-6*, Myh6* α-myosin heavy chain*, Myh7* β-myosin heavy chain*, Nos2* inducible nitric oxide synthase*, Nox4* NADPH-oxidase type 4*, Nppa* A-type natriuretic peptide*, Nppb* B-type natriuretic peptide*, Ppia* Peptidyl prolyl isomerase A, *Tnf-α* tumor necrosis factor alpha. *Ppia* was used as a housekeeping gene for normalization.

### Both losartan and mirabegron attenuated the left ventricular fibrosis in CKD

The development of left ventricular fibrosis in CKD was investigated by quantifying picrosirius red and fast green stained slides. Significant interstitial fibrosis was found in the CKD group compared to the sham-operated group (Fig. 4e,f). Losartan treatment significantly decreased the left ventricular fibrosis compared to the CKD group, probably, due to its well-known antiremodeling effects by blocking the AT1 receptor. Collagen deposition was not significantly different in the mirabegron-treated CKD group as compared to the sham-operated group; however, it showed a trend toward a decrease as compared to the CKD group (p = 0.067) (Fig. 4e,f). To further strengthen our results, the LV expression of fibrosis and heart failure markers were measured by qRT-PCR. In the CKD group, the LV expression of the collagen type I alpha 1 (*Col1a1*) failed to increase significantly as compared to the sham-operated group. However, another fibrosis marker in the angiotensin-II/TGF-β pathway, the connective tissue growth factor (*Ctgf*) was up-regulated significantly compared to the sham-operated group, indicating an active fibrotic process in CKD at week 13 (Table 3). Neither losartan nor mirabegron reduced the LV expression of *Col1a1* significantly as compared to the CKD or sham-operated groups; however, it showed a tendency of decrease in the mirabegron-treated CKD group as compared to the CKD group. *Ctgf* showed significant repression in response to losartan as compared to the CKD group (Table 3). In contrast, *Ctgf* was significantly overexpressed in the mirabegron-treated CKD group compared to the CKD or the sham-operated groups (Table 3). These results also point out that mirabegron might ameliorate cardiac fibrosis via *Ctgf*-independent mechanisms in uremic cardiomyopathy. The left ventricular expression of the heart failure markers A-type and B-type natriuretic peptides (*Nppa* and *Nppb,* respectively) were not significantly different between the CKD and sham-operated groups, indicating that the CKD animals are rather in a compensated heart failure stage (Table 3). Losartan but not mirabegron-treatment resulted in a significant LV repression of *Nppa* and *Nppb* compared to the CKD group (Table 3).

### Both losartan and mirabegron repress the cardiac inflammatory and nitro-oxidative stress markers in uremic cardiomyopathy at the transcript level

Inflammatory processes triggered by elevated nitro-oxidative stress are major contributors to the development of uremic cardiomyopathy. The overexpression of the superoxide anion producing nicotinamide adenine dinucleotide phosphate-oxidase (NADPH-oxidase or *Nox)* isoforms and inducible nitric oxide synthase (iNOS or *Nos2*) are major sources of elevated nitro-oxidative stress in the heart^6^. In the CKD group, LV *Nox4* expression showed a statistically non-significant increase by 35% (p = 0.078), and *Nos2* expression increased significantly compared to the sham-operated group (Table 3). Both losartan and mirabegron treatment significantly reduced the LV *Nox4* and *Nos2* expression compared to the CKD or sham-operated groups (Table 3). The LV expressions of the inflammatory cytokines interleukin-1 (*IL-1)*, interleukin-6 (*IL-6*), and tumor necrosis factor-α (*Tnf-α*) were significantly increased in the CKD group as compared to the sham-operated group, indicating tissue inflammation (Table 3). Both losartan and mirabegron caused significant repression in the *IL-6* mRNA levels compared to the CKD group (Table 3). Furthermore, losartan but not mirabegron decreased the expression of *IL-1* and *Tnf-α* significantly as compared to the CKD group (Table 3). Angiotensin-II is known to increase the nitro-oxidative stress and inflammation via AT1 (*Agtr1a*) receptors by increasing NADPH-oxidase levels and inflammatory markers such as IL-6 and TNF-α^28^. Interestingly, LV *Agtr1a* was significantly repressed in the CKD group compared to the sham-operated group, probably as a compensatory mechanism of the chronically over-activated RAAS in CKD (Table 3). The losartan-treated CKD group showed no significant difference in the LV *Agtr1a* expression compared to the CKD or sham-operated groups. However, mirabegron-treatment significantly decreased the LV *Agtr1a* expression as compared to CKD or sham-operated groups (Table 3). There was no significant difference in the LV angiotensinogen (*Agt*) expression at the mRNA level between the groups (Table 3).

### β3-AR expression did not increase in uremic cardiomyopathy irrespective of losartan or mirabegron-treatment

**Figure 5:**
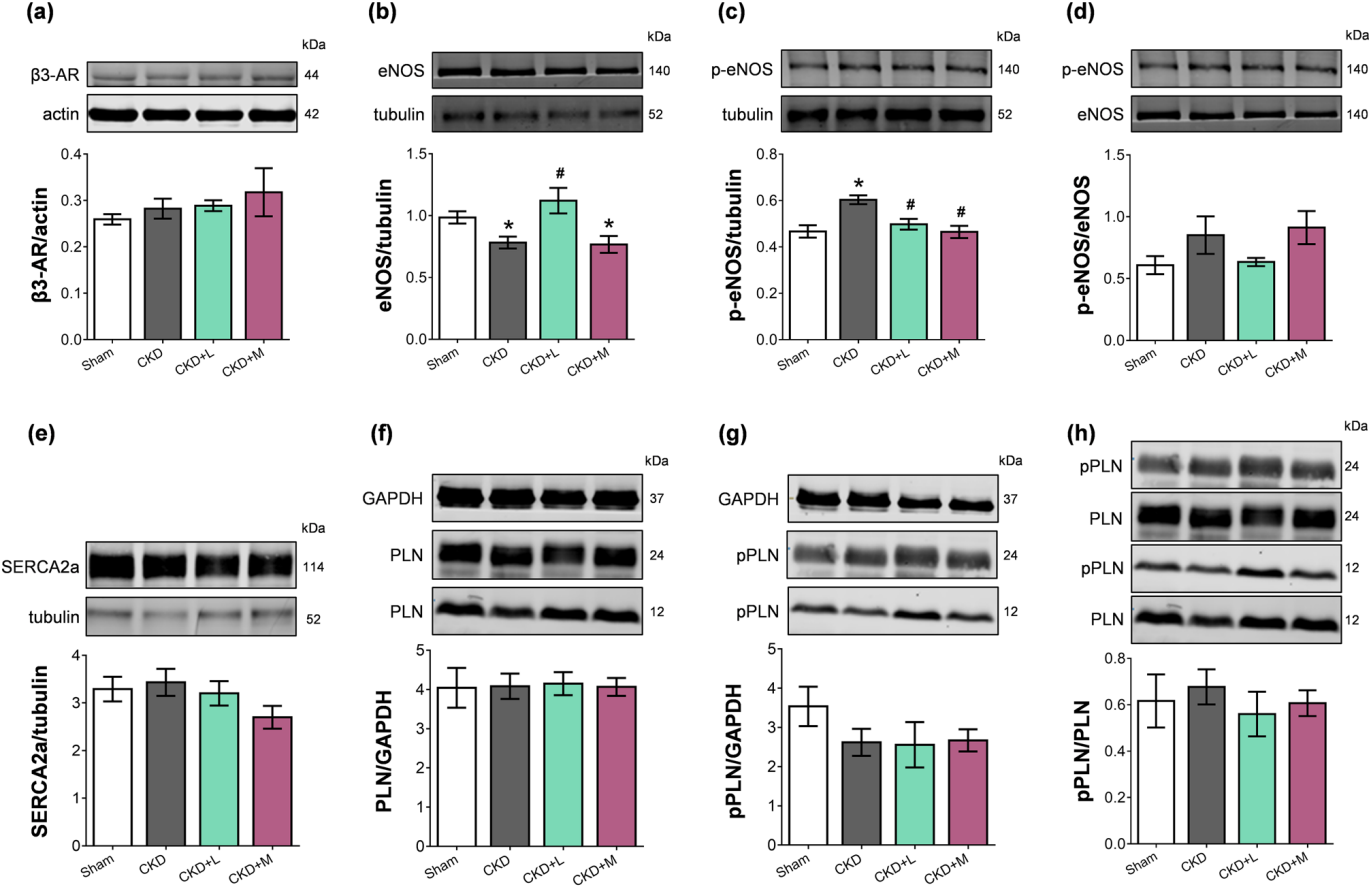
The effects of losartan and mirabegron on the protein expression at week 13 assessed by Western blot. Left ventricular expression and cropped representative images of (**a**) beta-3 adrenergic receptor (β3-AR), (**b**) endothelial nitric oxide synthase (eNOS), (**c**) phospho-eNOS, (**d**) phospho-eNOS/ eNOS ratio, (**e**) sarcoendoplasmic reticulum calcium ATPase 2a (SERCA2a), (**f**) phospholamban (PLN), (**g**) phospho-PLN (pPLN), and (**h**) phospho-PLN/PLN ratio. Values are presented as mean ± S.E.M., *p < 0.05 vs. sham-operated group, ^#^p < 0.05 vs. CKD group (n = 6–7, One-Way ANOVA, Holm-Sidak post hoc test). *Sham* sham-operated group, *CKD* chronic kidney disease group, *CKD + L* losartan-treated chronic kidney disease group, *CKD + M* mirabegron-treated chronic kidney disease group. Images were captured with the Odyssey CLx machine and exported with Image Studio 5.2.5 software. The full-length Western blots are presented in Supplementary Figures S1-12.

Myocardial overexpression of the β3-AR was reported in heart failure of different etiology^14^. LV β3-AR (*Adrb3*) mRNA expression showed only an increasing tendency in the CKD group compared to the sham-operated group if analyzed by One-Way ANOVA in the four groups (Table 3). Notably, if only the sham-operated and the CKD groups were compared by unpaired t-test, the left ventricular β3-AR mRNA expression was significantly increased in the CKD group compared to the sham-operated group (0.99 ± 0.07 vs. 0.65 ± 0.10 relative gene expression, p = 0.012). However, β3-AR protein levels failed to increase in the CKD group compared to the sham-operated group (Fig. 5a, Supplementary Fig. S1, and S2). Neither losartan nor mirabegron changed the β3-AR mRNA or protein levels significantly in uremic cardiomyopathy (Table 3, Fig. 5a).

### Losartan but not mirabegron increased the eNOS protein level in uremic cardiomyopathy

Decreased activity of endothelial NOS (eNOS) and nitric oxide (NO) bioavailability are considered major contributors to HFpEF and uremic cardiomyopathy^29,30^. Accordingly, the LV expression of eNOS protein was significantly decreased in the CKD group compared to the sham-operated group in our model (Fig. 5b). Losartan treatment significantly increased the eNOS level; however, mirabegron treatment could not increase it (Fig. 5b, Supplementary Fig. S3, and S4). Phospho-eNOS protein level was significantly increased, but phospho-eNOS/eNOS ratio did not increase significantly in the CKD group compared to the sham-operated group (Fig. 5c,d). Phospho-eNOS levels were not significantly different in losartan or mirabegron-treated CKD groups compared to the sham-operated group and were significantly decreased compared to the CKD group (Fig. 5c, Supplementary Fig. S5, and S6). There was no significant difference in the phospho-eNOS/eNOS ratio among the groups (Fig. 5d).

### No changes in SERCA2a and phospholamban protein levels in uremic cardiomyopathy irrespective of losartan or mirabegron-treatment

Decreased SERCA2a expression and disturbed calcium homeostasis are related to impaired diastolic relaxation and declining systolic function^31^. In contrast, the SERCA2a protein level was not different in the experimental groups in our present study (Fig. 5e, Supplementary Fig. S7, and S8). The phosphorylation of phospholamban (PLN) can increase the SERCA activity and improve the speed of muscle relaxation^31^. However, there was no difference in the PLN expressions between the groups (Fig. 5f, Supplementary Fig. S9, and S10). Notably, phospho-PLN (pPLN) protein levels showed a tendency of decrease in the CKD and mirabegron-treated CKD groups compared to the sham-operated group (p = 0.157 and p = 0.137, respectively, unpaired t-test) (Fig. 5g, Supplementary Fig. S11, and S12). There was no significant difference in phospho-PLN protein level between the losartan-treated CKD and sham-operated or CKD groups (Fig. 5g). The phospho-PLN/PLN ratios did not decrease in the CKD group, and losartan or mirabegron did not change the phospho-PLN/PLN ratio in CKD (Fig. 5h).

## Discussion

In the 5/6th nephrectomized rats, uremic cardiomyopathy developed with similar laboratory and cardiac alterations to those in CKD-induced HFpEF patients. Our present study demonstrates first that there is no difference in the left ventricular β3-AR expression at the protein level in the HFpEF phase of uremic cardiomyopathy in rats. Here we also demonstrate for the first time in the literature that the β3-AR agonist mirabegron has no anti-hypertrophic but mild anti-fibrotic and anti-inflammatory effects in uremic cardiomyopathy. These anti-fibrotic and anti-inflammatory effects seem to be independent of the β3-AR coupled eNOS-mediated pathways and might be explained by the effect of mirabegron causing repression on AT1 receptor in uremic cardiomyopathy. The ARB losartan significantly ameliorated the development of uremic cardiomyopathy via anti-hypertrophic, anti-fibrotic, anti-inflammatory, and eNOS-associated mechanisms.

Our present findings on the characteristic laboratory and echocardiographic parameters in CKD are consistent with the literature data and our previous results in 5/6th nephrectomized rats^32–35^. Our previous study showed no significant differences in the baseline laboratory and echocardiographic results between the sham-operated and CKD animals^35^. Therefore, we did not characterize the baseline (i.e., pre-operative) laboratory and echocardiographic parameters in our present study. In our present study, week 4 laboratory and echocardiographic parameters confirmed that 5/6th nephrectomized animals developed renal failure at the same severity in each CKD group before starting the drug administrations. We have found here characteristic laboratory changes in CKD, including increased serum urea and creatinine concentrations and urine protein levels and decreased creatinine clearance 13 weeks after the operations. Moreover, renal anemia, hypercalcemia, and hypercholesterolemia developed in the 5/6th nephrectomized animals. In our CKD model, echocardiography confirmed the presence of uremic cardiomyopathy characterized by LVH and DD. Histology confirmed LVH at the cellular level and revealed LV fibrosis. According to these findings, the severity of CKD in our model corresponds to the human G2 or G3a CKD stages with mildly or moderately decreased kidney function^36,37^. Notably, severe hypertension is usually not a feature of the 5/6th nephrectomy-induced CKD models^34^. Indeed, our CKD model showed only a tendency of increase in blood pressure values. Therefore, DD might be developed due to LVH and fibrosis without severe hypertension.

Both pre-clinical and clinical studies showed that CKD-specific and non-specific risk factors such as uremic toxins, renal anemia, the over-activation of the RAAS and sympathetic nervous system, elevated nitro-oxidative stress, decreased NO levels, increased systemic and tissue inflammation could provoke the development of uremic cardiomyopathy^6,7^. Indeed, our CKD model showed significantly elevated white blood cell count and overexpressed pro-inflammatory cytokines, including *IL1*, *IL6, and Tnf-α* in the left ventricle, indicating systemic and tissue inflammation. The interplay of increased nitro-oxidative stress and inflammation can be an essential contributor to LVH in CKD^3,6,7^ and was linked to the progression of HFpEF^30^. NOX, producing superoxide anion, may also be involved in the development of cardiac hypertrophy, interstitial fibrosis, or contractile dysfunction^38^. Accordingly, LV *Nox4* expression showed a statistically non-significant increase in our CKD model. Reduced NO bioavailability due to increased superoxide levels and consequential peroxynitrite formation might contribute to cardiomyocyte stiffness and fibrosis, leading to diastolic dysfunction and microvascular endothelial dysfunction in HFpEF and uremic cardiomyopathy^39^. Our CKD model showed a decreased myocardial eNOS protein level, probably, due to the capillary rarefaction and hypertrophy in CKD, and a significantly elevated eNOS phosphorylation on the main activation site (Ser1177). The latter could be a compensatory mechanism to increase NO production in uremic cardiomyopathy. Accordingly, the p-eNOS/eNOS ratio was tendentiously increased in CKD as compared to the sham operated-group. Our results on eNOS expression and activity in uremic cardiomyopathy are in line with the literature data^40^. Furthermore, eNOS uncoupling leads to ROS/RNS instead of NO production, increasing the nitro-oxidative stress and hypertrophic remodeling^39,41^. According to studies using animal models and human myocardial samples, the up-regulation of iNOS (i.e., *Nos2*) can contribute to the dysregulation of NO production in oxidative and pro-inflammatory states leading to increased peroxynitrite production, which can be associated with pathologic cardiac remodeling^30,41^. Indeed, in our present study, *Nos2* expression was significantly increased in uremic cardiomyopathy. On the other hand, eNOS-derived NO production was demonstrated to enhance LV relaxation via PLN-mediated SERCA Ca^2+^ re-uptake^29^. Decreased SERCA2a expression and disturbed calcium homeostasis could also be associated with impaired relaxation and declining systolic function in later stages of HF^31^. In contrast, Silveira et al*.* found that SERCA2a and its regulator PLN did not decrease in early isolated diastolic dysfunction induced by aortic stenosis^42^. Primessnig et al. demonstrated in 5/6th-nephrectomy-induced CKD in rats that reduced phosphorylation of PLN could lead to reduced SERCA activity 8 weeks after the operation. However, they found increased PLN phosphorylation 24 weeks after the operation, pointing out the time-dependency of PLN phosphorylation and SERCA activity in uremic cardiomyopathy^43^. In our study, there was no significant change in SERCA2a and PLN expressions 13 weeks after the operations in CKD. These results might be explained by the longer follow-up time in our study than the 8-week follow-up period used in the study of Primessnig et al*.*^43^. Losartan and mirabegron did not change the SERCA expression and pPLN/PLN expressions in CKD, suggesting that these proteins did not play a crucial role in the effects causing improved diastolic function in the losartan and mirabegron-treated groups.

Most of the standard therapies in uremic cardiomyopathy are supportive and used to modulate comorbidities or heart failure slowing down the progression of the condition^44^. RAAS inhibitors, including ARBs, are cornerstone therapies to reduce proteinuria, CKD progression, and cardiovascular risk^45^. The ARB losartan was reported to ameliorate LVH and myocardial fibrosis development and improve cardiac function in 5/6th nephrectomized rats and end-stage renal disease patients^46,47^. In our present study, losartan failed to improve the main routine laboratory parameters of kidney function, including creatinine clearance, serum carbamide, and creatinine levels. However, it reduced the urine volume and the remnant kidney weight compared to the CKD group showing a mild renoprotective effect. The further histological or molecular examination of the kidneys was out of the scope of the present study.

Evidence suggests that chronic over-activation of the RAAS and the sympathetic nervous system in HF also stimulates the inflammatory processes and increases nitro-oxidative stress, further aggravating each other^48,49^. Angiotensin-II has been reported to activate cardiac NADPH-oxidase via AT1 receptor and, subsequently, the over-production of ROS/RNS. The increased nitro-oxidative stress could trigger the production of pro-inflammatory mediators, such as IL1, IL6, TNF-α, and tissue growth factor-beta (TGF-β)^49,50^, and the inflammatory cytokines could decrease eNOS expression and NO bioavailability, contributing to cardiac remodeling and HF^28^. Therefore, AT1 blockade by losartan is a rational therapeutic option to ameliorate cardiac remodeling by reducing nitro-oxidative stress, inflammatory processes and improve NO bioavailability in uremic cardiomyopathy. Indeed, in our present study, losartan improved the DD, LVH, and cardiac fibrosis, probably via blocking the AT1 receptor-mediated nitro-oxidative (*Nox4* and *Nos2*) and inflammatory (*IL1, IL6,* and *Tnf-α*), and eNOS-associated mechanisms in our CKD model. Interestingly, it has been reported that another ARB, valsartan combined with the neprilysin inhibitor sacubitril (LCZ696) attenuated cardiac hypertrophy and fibrosis in 5/6th nephrectomized rats^51^ and in human phase I clinical trials in advanced CKD patients with HF (clinical trial No.: NCT03771729 and NCT04218435). Taken together, our present results are in line with the literature data on the antiremodeling effects of ARBs in uremic cardiomyopathy.

Modulation of NO signaling is considered one of the novel promising approaches in HFpEF treatment^41^. β3-AR agonists are reported to ameliorate cardiac remodeling, diastolic and systolic dysfunction via the β3-AR receptor coupled eNOS-derived NO production beyond other mechanisms such as preserving the function of the Na^+^/K^+^-ATP-ase, antioxidant effects via the AKT-NO-mPTP signaling, and influencing the fatty acid metabolism^14,39,41,52^. Currently, phase II and III clinical trials are investigating the antiremodeling effects of the β3-AR agonist mirabegron on the development of LVH and HFpEF (NYHA I-II stages, clinical trial No.: NCT02599480), and HFrEF (NYHA III-IV stages, clinical trial No.: NCT01876433 and NCT03926754). Notably, kidney function is often decreased in HFpEF and HFrEF patients, and chronic heart failure aggravates renal dysfunction mutually. This bidirectional interaction of renal and heart failure is the key concept in cardio-renal syndrome^53,54^. Dysfunction of each organ can induce and perpetuate injury in the other via complex hemodynamic, neurohormonal, and biochemical pathways such as over-activated RAAS and sympathetic nervous system, increased nitro-oxidative stress, and inflammatory pathways^6^. Notably, patients with uncontrolled hypertension, severe anemia, moderately or severely decreased renal function (eGFR < 30 or 50 mL/min corresponding to G3b-G5 CKD stages, respectively) were excluded from the aforementioned phase II and phase III clinical trials. Thus, to our best knowledge, the antiremodeling effects of mirabegron were not investigated in a selected patient population suffering from uremic cardiomyopathy. Considering the exclusion criteria of the aforementioned clinical trials, we aimed to investigate the effects of mirabegron in mild to moderate experimental uremic cardiomyopathy. In our study, mirabegron did not affect the major parameters of renal function, including serum carbamide and creatinine levels, creatinine clearance, and urine volume at the endpoint. However, it had several adverse effects on glomerular function, worsening the proteinuria, renal anemia, serum total cholesterol, and LDL-cholesterol levels in our CKD model. Although mirabegron repressed the left ventricular *IL6* level, white blood cell count remained elevated, suggesting the presence of low-grade systemic inflammation in CKD irrespective of mirabegron-treatment.

In our present study, mirabegron could not prevent the development of left ventricular hypertrophy assessed by M-mode echocardiography and histology in CKD. We speculate that the failing β3-AR overexpression and the β3-AR-coupled activation of eNOS-mediated pathways are responsible for its missing anti-hypertrophic effects in CKD. Notably, if only the sham-operated and CKD groups were compared by unpaired t-test, the left ventricular β3-AR mRNA expression was significantly increased in the CKD group compared to the sham-operated group (p = 0.012, Table 3). However, the protein level showed no difference between the groups (p = 0.336, Fig. 5a). This could be explained by (i) increased degradation and turnover rate of proteins in CKD as it is considered as a catabolic state^55^, and (ii) the β3-AR expression could be regulated time-dependently in uremic cardiomyopathy. We also measured the left ventricular β3-AR mRNA and protein levels at an earlier follow-up time point in the same CKD model 5/6 nephrectomy-induced CKD in male Wistar rats 9 weeks after the operations (Supplementary Fig. S13). At this time, HFpEF is also developed, as we described previously^35^. At this earlier follow-up time point, the left ventricular expression of β3-AR mRNA was also significantly increased in the CKD group as compared to the sham-operated group, and there was no significant change in the β3-AR protein level between the groups, similarly to the week 13 findings (Supplementary Fig. S13). However, it could not be excluded that the β3-AR protein expression might change in a later phase of uremic cardiomyopathy. Notably, Miao et al*.* demonstrated in aortic banding-induced heart failure in rats that the cardiac expression of β3-AR mRNA and protein expressions in myocardial tissues showed a positive correlation with aging and the severity of heart failure^56^.

Most studies investigating the effects of β3-AR agonists in heart failure demonstrated that β3-AR agonists attenuated cardiac hypertrophy and fibrosis and improved cardiac contractility via coupling of β3-AR to the eNOS/cGMP pathway as the main mechanism^14,52^. Therefore, we also investigated the left ventricular expression of eNOS, p-eNOS, and their ratio in our study. Mirabegron did not increase the total eNOS level and significantly decreased the p-eNOS level as compared to the CKD group. Therefore, it seems that the β3-AR is uncoupled from eNOS or unable to activate it, probably, due to elevated nitro-oxidative stress, inflammatory mechanisms, and uremic toxins-induced alterations^40^. The precise mechanistic characterization of CKD-induced changes of the β3-AR-mediated pathways was out of the scope of the present study.

However, in our present study, mirabegron had beneficial effects on DD characterized by e’ and E/e’, and cardiac fibrosis assessed by histology and left ventricular expressions of *Col1a1* and *Ctgf*. We hypothesized that mirabegron could have its antiremodeling effects independently of the β3-AR-eNOS-mediated pathway in uremic cardiomyopathy. Interestingly, mirabegron reduced the left ventricular expression of the AT1 receptor in uremic cardiomyopathy. This finding is particularly interesting since the chronic administration of the β3-AR agonists BRL37344 could down-regulate the AT1 receptor expression and up-regulate the AT2 receptor expression in the pancreatic^23^ and lung tissues^24^ of male apoE knock-out mice. Interestingly, Belge et al*.* demonstrated that angiotensin-II administration did not induce cardiac fibrosis and hypertrophy in mice with cardiomyocyte-specific expression of human β3-AR^22^. Recently, Kamiya et al*.* demonstrated that chronic infusion of a β3-adrenergic receptor agonist attenuated cardiac fibrosis and improved diastolic dysfunction independently of blood pressure in an angiotensin-II-induced heart failure model with hypertension in mice^57^. These results suggest that chronic β3-adrenoceptor activation regulates the expression of angiotensin-II receptor types, and these interactions may play a protective role in lung, pancreatic, and other tissues such as the heart. The chronic over-activation of the AT1 receptor could play a major role in oxidative stress, inflammation, and ultimately fibrosis in the development of heart failure^58,59^. Indeed, in our present study, mirabegron significantly reduced the left ventricular expression of the nitro-oxidative stress markers *Nox4* and *Nos2* and the inflammatory marker *IL6* in uremic cardiomyopathy. Our present results are in line with these literature data since mirabegron treatment significantly reduced the AT1 receptor expression in uremic cardiomyopathy, which might lead to its antioxidant, anti-inflammatory, and anti-fibrotic effects independently of the eNOS/cGMP-mediated pathway in uremic cardiomyopathy. Moreover, Hadi et al*.* demonstrated the dual antioxidant role of β3-ARs in macrophages: (i) β3-AR decreases ROS production by directly inhibiting NADPH-oxidase activity; and (ii) β3-AR induces the expression of catalase, a major hydrogen peroxide scavenger^58^. They also confirmed that β3-AR stimulation inhibited the ROS-dependent nuclear factor kappa-light-chain-enhancer of activated B cell (NF-κB) inflammatory pathway in the uterus^58^. In another study, β3-AR stimulation inhibited cytokine production, including TNF-α and IL6, to prevent myometrial cell apoptosis and extracellular matrix remodeling^59^. However, these secondary antioxidant and anti-inflammatory mechanisms of the β3-AR induction by mirabegron are not well-characterized in HF. Our present finding on the antiremodeling effects of mirabegron independently of β3-AR-eNOS mediated pathways could be interesting for physicians treating heart failure patients with reduced renal function since mirabegron could have anti-fibrotic effects in the early stages of uremic cardiomyopathy. Therefore, the involvement of early-stage CKD patients in clinical studies investigating the effects of mirabegron could be considered in the future.

In summary, our present study suggests that the development of uremic cardiomyopathy might be prevented or markedly slowed down by losartan if its administration starts in early CKD stages without manifest LVH. In contrast, mirabegron failed to improve the severity of LVH, but had several beneficial effects on diastolic dysfunction, fibrosis, and tissue inflammation in uremic cardiomyopathy. These antiremodeling effects seem to be independent of the β3-AR/eNOS mediated pathways in uremic cardiomyopathy and probably associated with the effects of mirabegron downregulating the AT1 receptor. Mirabegron had adverse effects on glomerular function with worsening anemia, proteinuria, and serum cholesterol levels in our CKD model. Therefore, the elevated mortality risk and the possibility of future cardiovascular events might remain high in CKD patients treated by mirabegron. In future studies, the combination of mirabegron with ARBs or cholesterol-lowering drugs such as statins might be rational to potentiate the antiremodeling effects and prevent the adverse effects of mirabegron.

## Limitations

The purpose of this study was to evaluate and compare the effects of chronic administration of ARB losartan and β3-AR agonist mirabegron on cardiac remodeling, function, and the development of HFpEF in uremic cardiomyopathy in rats. Uremic cardiomyopathy is a unique entity most commonly manifested as left ventricular hypertrophy with preserved ejection fraction and predisposing the heart for further cardiovascular complications in CKD^60^. However, there are still many unknown mechanisms, such as the exact role of β3-AR in the development of uremic cardiomyopathy. We demonstrated here that there is no difference in the left ventricular β3-AR expression at the protein level 13 weeks after the 5/6th nephrectomy in rats. However, it could not be excluded that the β3-AR protein expression might change in a later HFrEF phase of uremic cardiomyopathy. The time-dependent and mechanistic investigation of the cardiac β3-AR expression in the development of uremic cardiomyopathy was out of the scope of the present study. Notably, the precise mechanisms and functional role of the β3-AR in heart failure induced by different cardiovascular diseases, including diabetes mellitus, acute myocardial infarction, or chemotherapy-induced heart failure forms, are also not fully discovered. Therefore, we focused mainly on the potential protective effects of mirabegron in CKD, investigating the well-known markers of uremic cardiomyopathy and the effects caused by mirabegron. The deep mechanistic insight of the antiremodeling effects of mirabegron was out of the scope of our present descriptive study. We found here moderate antiremodeling effects of mirabegron and hypothesized that mirabegron could have anti-fibrotic effects in uremic cardiomyopathy independently of the β3-AR-eNOS-mediated pathway, but probably associated with its effects causing repression on the AT1 receptor. However, further mechanistic studies are needed to explore the antiremodeling effects of mirabegron in uremic cardiomyopathy.

## Materials and methods

This investigation conformed to the National Institutes of Health Guide for the Care and Use of Laboratory Animals (NIH Publication No. 85–23, Revised 1996) and was approved by the Animal Research Ethics Committee of Csongrád County (XV./799/2019) and the University of Szeged in Hungary. All institutional and national guidelines for the care and use of laboratory animals were followed. The authors complied with the ARRIVE guidelines.

### Animals

In this study, a total of 45 adult male Wistar rats (*Rattus norvegicus*, 8 weeks old, 280–340 g) were housed in pairs in individually ventilated cages (Sealsafe IVC system, Tecniplast S.p.A., Italy). The rats were maintained with 12 h:12 h light/dark cycles in a temperature-controlled room throughout the study. Standard rat chow and tap water were supplied ad libitum.

### Experimental setup

Control animals underwent sham-operation (n = 10), and experimental CKD (n = 35) was induced by 5/6th nephrectomy in two phases. After the operations, rats were followed-up for 13 weeks (Fig. 1). On the first day of the 5th follow-up week, rats were divided into the following four groups and treated via oral *gavage* daily for 8 weeks: (1) sham-operated group treated with *per os* tap water (n = 10, 2 mL/kg/day), (2) CKD group treated with *per os* tap water (n = 13, 2 mL/kg/day), (3) CKD group treated with *per os* losartan (n = 12, 10 mg/kg/day dissolved in tap water in 2 mL/kg end volume, Arbartan 50 mg film-coated tablets, Teva Pharmaceutical Industries Ltd., Hungary), and (4) CKD group treated with *per os* Mirabegron (n = 10, 10 mg/kg/day in tap water 2 mL/kg end volume; Betmiga 50 mg prolonged-release tablets, Astellas Pharma Europe B.V., Netherland) (Fig. 1). In our study, 6 animals died from the nephrectomized groups (2 animals in the CKD group, 1 animal in the losartan-treated CKD group, and 3 animals in the mirabegron-treated CKD group). At weeks 4 and 12, cardiac morphology and function were assessed by transthoracic echocardiography (Fig. 1). Blood was collected from the saphenous vein at week 4 and from the abdominal aorta at week 13 to measure serum parameters. The animals were placed into metabolic cages (Tecniplast S.p.A., Italy) for 24 h at weeks 4 and 12 to measure urine creatinine and protein levels (Fig. 1). At week 13, invasive blood pressure measurements were performed in a subgroup of animals (Fig. 1) (n = 6–8). At week 13, hearts were isolated, then left ventricular samples were prepared for histology and biochemical measurements. The development of LVH and fibrosis in CKD was verified by the measurement of cardiomyocyte diameters and cross-sectional areas on hematoxylin–eosin (HE) stained slides and picrosirius red/fast green-stained (PSFG) slides. Total RNA was isolated from the left ventricles, and the myocardial expression of *Adrb3*, *Agt, Agtr1a, Col1a1, Ctgf, IL1, IL6, Myh6, Myh7, Nos2, Nox4, Nppa, Nppb,* and *Ppia* were measured by qRT-PCR. Moreover, LV expression of β3-AR, total-eNOS, phospho-eNOS, PLN, phospho-PLN, and SERCA2a were measured by Western blot.

### 5/6th nephrectomy

Sham operation and 5/6th nephrectomy were performed in two phases as described previously^33,35^ (Fig. 1). Briefly, anesthesia was induced by intraperitoneal injection of pentobarbital sodium (Euthasol, Produlab Pharma B.V., Netherlands; 40 mg/kg). At the first operation, the 1/3 left kidney on both ends was excised. One week after the first operation, animals were anesthetized again, and the right kidney was removed. During sham operations, only the renal capsules were removed. After the surgeries, the incision was closed with running sutures, and povidone-iodine was applied on the surface of the skin. As a postoperative medication, *sc.* 0.3 mg/kg nalbuphine hydrochloride (Nalbuphine 10 mg/ml, Teva Pharmaceutical Industries Ltd., Hungary) was administered for 4 days, twice in the first two postoperative days and once in the third and fourth post-operative days. Enrofloxacin antibiotics (Enroxil 75 mg tablets, Krka, Slovenia; dissolved in tap water in 3.5 mg/L end concentration) were administered in the drinking water for 4 days after both surgeries.

### Transthoracic echocardiography

Cardiac morphology and function were assessed by transthoracic echocardiography at weeks 4 and 12 as we described previously^35,61–63^ (Fig. 1). Rats were anesthetized with 2% isoflurane (Forane, Aesica, Queenborough Limited, UK). Two-dimensional, M-mode, Doppler, tissue Doppler, and 4 chamber-view images were performed by the criteria of the American Society of Echocardiography with a Vivid IQ ultrasound system (General Electric Medical Systems, USA) using a phased array 5.0–11 MHz transducer (GE 12S-RS probe). Data of three consecutive heart cycles were analyzed (EchoPac Dimension v201, General Electric Medical Systems, USA; https://www.gehealthcare.com/products/ultrasound/vivid/echopac) by an experienced investigator in a blinded manner. The mean values of three measurements were calculated and used for statistical evaluation.

### Blood pressure measurement

To measure arterial blood pressure in a separated subgroup of animals, a PE50 polyethylene catheter (Cole-Parmer, USA) was inserted into the left femoral artery at week 13 under pentobarbital anesthesia (Euthasol, Produlab Pharma B.V., Netherlands; 40 mg/kg) as described previously^35,64^. Blood pressure measurements were performed between 09:00 and 14:00 h.

### Serum and urine laboratory parameters

Serum carbamide and creatinine levels were quantified by kinetic UV method using urease and glutamate dehydrogenase enzymes and Jaffe method, respectively. Serum sodium, potassium, and chloride levels were determined by indirect potentiometry using ion-selective electrodes. The serum calcium, magnesium, phosphate levels were quantified by complex formation methods. All reagents and instruments for the serum parameter measurements were from Roche Diagnostics (Hoffmann-La Roche Ltd, Switzerland), as described previously^33,35^. Urine creatinine and urine protein levels were measured by standard laboratory methods as described previously^33,35^. At week 13, serum total cholesterol, HDL-cholesterol and triglyceride levels were measured by Roche Cobas 8000 analyzer system using enzymatic colorimetric assays from Roche (Hoffmann-La Roche Ltd, Switzerland)^65,66^. LDL-cholesterol was calculated according to the Friedewald formula. Total blood count and hematocrit were measured from whole blood by a hematology analyzer (XE-2100, Sysmex Corporation, Japan) at week 13 to verify the development of chronic systemic inflammation and renal anemia.

### Tissue harvesting

At week 13, hearts were isolated under pentobarbital anesthesia, and the blood was washed out in calcium-free Krebs–Henseleit solution. Then the hearts were weighed, left and right ventricles were separated and weighed, and the cross-section of the left ventricle at the ring of the papillae was cut and fixed in 4% buffered formalin for histological analysis. Other parts of the left ventricles were freshly frozen in liquid nitrogen and stored at − 80 °C until further biochemical measurements. Body weight, tibia length, left kidney weight, and weight of lungs were measured at week 13.

### Hematoxylin–eosin and picrosirius red and fast green stainings

5 μm paraffin-embedded transverse cut sections of the formalin-fixed subvalvular areas of the left ventricles were stained with hematoxylin–eosin (HE) or picrosirius red and fast green (PSFG) as described previously^35,62,63^. Histological slides were scanned with a Pannoramic Midi II scanner (3D-Histech, Hungary). On the digital HE images, cardiomyocyte diameters and cross-sectional areas were measured to verify the development of LVH at the cellular level. For the evaluation, the Biology Image Analysis Software (BIAS) was used^67^. BIAS (internal built, dated December 2019; https://single-cell-technologies.com/bias/) is developed by Single-Cell Technologies Ltd., Hungary and the first publicly available version expected to be released in late 2021. Image pre-processing was followed by deep learning-based cytoplasm segmentation. User-selected objects were forwarded to the feature extraction module, configurable to extract properties from the selected cell components. Here, transverse transnuclear cardiomyocyte diameter was measured in 100 selected, longitudinally oriented, mono-nucleated cardiomyocytes on left ventricular sections cut on the same plane. Cardiomyocyte cross-sectional areas of the same cells were calculated by the software as well. Cardiac fibrosis was assessed on PSFG slides with an in-house developed program^35,62,63^. Representative HE- and PSFG-stained slides were captured in Panoramic Viewer 1.15.4 (3D-Histech, Hungary; https://old.3dhistech.com/pannoramic_viewer).

### mRNA expression profiling by qRT-PCR

Quantitative RT-PCR was performed with gene-specific primers to monitor mRNA expression. RNA was isolated using QIAGEN RNeasy Fibrous Tissue Mini Kit (QIAGEN, Germany) from heart tissue. Then 100 μg of total RNA was reverse transcribed using iScript cDNA Synthesis Kit (Bio-Rad Laboratories Inc., USA). Specific primers (*Adrb3*: β-3 adrenergic receptor, #qRnoCED0006313; *Agt*: angiotensinogene, #qRnoCED0051666; *Agtr1a*: angiotensin-II receptor type 1, #qRnoCID0052626; *Col1a1*: collagen type 1 alpha 1 chain, #qRnoCED0007857; *Ctgf*: connective tissue growth factor, #qRnoCED0001593; *IL1*: interleukin-1, #qRnoCID0002056; *IL6*: interleukin-6, #qRnoCID0053166; *Myh6*: α-myosin heavy chain, #qRnoCID0001766; *Myh7*: β-myosin heavy chain, #qRnoCED0001215; *Nos2*: inducible nitric oxide synthase, #qRnoCID0017722; *Nox4*: NADPH-oxidase type 4, #qRnoCID0003969; *Nppa*: A-type natriuretic peptide, #qRnoCED0006216; *Nppb*: B-type natriuretic peptide, #qRnoCED0001541; *Ppia*: peptidyl-prolyl isomerase A, #qRnoCID0056995; Tnf-α: tumor necrosis factor-α, #qRnoCED0009117) and SsoAdvanced Universal SYBR Green Supermix (BioRad Laboratories Inc., USA) were used according to the manufacturer's instructions. *Ppia* was used as a house keeping control gene for normalization.

### Western blot

Standard Western blot technique was used in the case of β3-AR with actin, eNOS, phospho-eNOS, SERCA2a with α-tubulin, and PLN, phospho-PLN with GAPDH loading background as described previously^68^ (Supplementary Fig. S1-13). Left ventricular samples (*n* = 28) were homogenized with an ultrasonicator (UP100H, Hielscher Ultrasonics GmbH, Germany) in Radio-Immunoprecipitation Assay (RIPA) buffer (50 mM Tris–HCl (pH 8.0), 150 mM NaCl, 0.5% sodium deoxycholate, 5 mM ethylenediamine tetra-acetic acid (EDTA), 0.1% sodium dodecyl sulfate, 1% NP-40; Cell Signaling Technology Inc., USA) supplemented with phenylmethanesulfonyl fluoride (PMSF; Sigma-Aldrich, USA), sodium orthovanadate (Na_3_VO_4_; Sigma-Aldrich, USA) and sodium fluoride (NaF; Sigma-Aldrich, USA). The crude homogenates were centrifuged at 15,000 × g for 30 min at 4 °C. After quantifying the supernatants' protein concentrations using the BCA Protein Assay Kit (Pierce Thermo Fisher Scientific Inc., USA), 25 μg of reduced and denaturized protein was loaded. Then sodium dodecyl-sulphate polyacrylamide gel electrophoresis (SDS-PAGE, 50 V, 4 h) was performed (6% gel in case of eNOS, phospho-eNOS and SERCA2a, 10% gel in case of β3-AR, and 12% gel in case of PLN, phospho-PLN,) followed by the transfer of proteins onto a nitrocellulose membrane (10% methanol in case of eNOS, phospho-eNOS and SERCA2a and 20% methanol in case of β3-AR, PLN and phospho-PLN, 35 V, 2 h). The efficacy of transfer was checked using Ponceau staining. The membranes were cut vertically and horizontally into parts corresponding to the molecular weights of β3-AR, eNOS, phospho-eNOS, SERCA, PLN, phospho-PLN, GAPDH, actin, and α-tubulin. Membranes were blocked for 1 h in 5% (w/v) bovine serum albumin (BSA, Sigma-Aldrich, USA) supplemented with Na_3_VO_4_ and NaF, and were incubated with primary antibodies in the concentrations of 1:1000 against eNOS (#32027S, Cell Signaling Technology Inc., USA^69^), PLN (#14562S, Cell Signaling Technology Inc., USA^70^), phospho-PLN (Ser16/Thr17, #8496S, Cell Signaling Technology Inc., USA^71^), SERCA2a (#4388S, Cell Signaling Technology Inc., USA^72^), actin (612,656, BD Bioscienses, USA^73^) and α-tubulin (#2144S, Cell Signaling Technology Inc., USA^74^) or 1:5000 against GAPDH (#2118, Cell Signaling Technology Inc., USA^75^) or 1:500 against phospho-eNOS (Ser1177, #9570S, Cell Signaling Technology Inc., USA^76^) and β3-AR (AB101095, Abcam PLC, UK^77^) overnight at 4 °C in 5% BSA. Then the membranes were incubated with IRDye 800CW Goat Anti-Rabbit and/or IRDye 680RD Goat Anti-Mouse secondary antibody (LI-COR Biosciences, USA, in the concentrations of 1:5000) for 1 h at room temperature in 5% BSA to detect proteins with similar molecular weight on the same membrane where it is applicable. Fluorescent signals were detected by Odyssey CLx machine (LI-COR Biosciences, USA), and digital images were captured with Image Studio 5.2.5 (LI-COR Biosciences, USA; https://www.licor.com/bio/image-studio/). Then images were analyzed and evaluated by Quantity One 4.4.0 (Bio-Rad Laboratories Inc., USA; https://www.bio-rad.com/en-hu/product/quantity-one-1-d-analysis-software?ID=1de9eb3a-1eb5-4edb-82d2-68b91bf360fb).

### Statistical analysis

Statistical analysis was performed using GraphPad Prism 6.01 for Windows (GraphPad Software Inc., USA; https://www.graphpad.com/scientific-software/prism/). All values are presented as mean ± S.E.M., p < 0.05 was accepted as a statistically significant difference. One-Way ANOVA was used to determine the statistical significance between all measured parameters within each time point. Two-way repeated-measures ANOVA was used to determine the effects of CKD and the treatments on serum, urine, and echocardiographic parameters between week 4 and endpoint follow-up data. Holm-Sidak test was used as a post hoc test. In cases of phospho-eNOS/eNOS, SERCA2a, and phospho-PLN Western blot results, unpaired t-test was also used to investigate the statistical significance between CKD vs. sham-operated groups or drug-treated CKD vs. CKD groups (p-values are mentioned in the text).

## Supplementary Information

Supplementary Information.

## Data Availability

The data generated and analyzed during the current study are available from the corresponding authors on a reasonable request.
