# Supplementary material for: Comparison of the antiremodeling effects of losartan and mirabegron in a rat model of uremic cardiomyopathy
Source: Sci Rep. 2021 Sep 1;11:17495. doi: 10.1038/s41598-021-96815-5 (PMC8410807; doi:10.1038/s41598-021-96815-5)
Supplement: Supplementary file 1 — Supplementary Information. [file 41598_2021_96815_MOESM1_ESM.pdf]

Supplementary Information

Comparison of the antiremodeling effects of losartan and mirabegron in a rat model of uremic cardiomyopathy

Zsuzsanna Z. A. Kovács[1], Gergő Szűcs[1], Marah Freiwan[1], Mónika G. Kovács[1], Fanni M. Márványkövi[1], Hoa Dinh[1], Andrea Siska[2], Katalin Farkas[2], Ferenc Kovács[3,4], András Kriston[3,4], Péter Horváth[3,4,5], Bence Kővári[6], Bálint Gábor Cserni[6], Gábor Cserni[6], Imre Földesi[2], Tamás Csont[1]*, Márta Sárközy[1]*

[1]MEDICS Research Group, *Department of Biochemistry, Interdisciplinary Center of Excellence, University of Szeged,* Dóm tér 9, Szeged, H-6720, Hungary
[2]*Department of Laboratory Medicine, Faculty of Medicine, University of Szeged,* Semmelweis utca 6, Szeged, H-6720, Hungary
[3]*Synthetic and Systems Biology Unit, Biological Research Centre, Eötvös Loránd Research Network,* Temesvári krt. 62, Szeged H-6726, Hungary
[4]*Single-Cell Technologies Ltd*, Temesvári krt. 62, Szeged, H-6726, Hungary
[5]*Institute for Molecular Medicine Finland (FIMM), University of Helsinki*, Helsinki 00014, Finland
[6]*Department of Pathology, Faculty of Medicine, University of Szeged*, Állomás utca 1, Szeged H-6720, Hungary

*Correspondence should be addressed to
sarkozy.marta@med.u-szeged.hu, ORCID ID: 0000-0002-5929-2146
csont.tamas@med.u-szeged.hu, ORCID ID: 0000-0001-5792-2768

Department of Biochemistry
Faculty of Medicine
University of Szeged
Dóm tér 9
Szeged, H-6720
Hungary
Tel.: +36/30-773-5811

Supplementary Table S1 Echocardiographic results at week 4

Parameter (unit)	Groups			
	Sham	CKD	CKD+losartan	CKD+mirabegron
EF (%)	58±1	57±1	56±1	59±2
HR (1/min)	372±8	367±12	363±11	362±12
LVEDV (µl)	131±7	136±5	137±7	136±6
LVESV (µl)	56±4	58±3	61±3	56±2
SV (µl)	75±4	78±3	76±4	82±7
CO (mL/min)	28±2	29±1	28±2	28±3
IVCT (ms)	14.5±1.02	14.8±0.65	14.57±0.43	15.86±1.1
IVRT (ms)	14.2±0.29	14.22±0.49	14.13±0.35	13.14±0.91
E-velocity (m/s)	1.06±0.05	1.04±0.04	1.02±0.06	0.99±0.05
e'-velocity (m/s)	0.05±0.003	0.05±0.003	0.05±0.004	0.05±0.003
E/e'	20.85±1.88	22.5±2.01	21.3±1.69	19.43±2.12
AWTs (mm)	3.2±0.1	3.35±0.13	3.31±0.14	3.35±0.09
AWTd (mm)	1.85±0.05	1.9±0.09	1.96±0.05	1.91±0.07
IWTs (mm)	3.31±0.11	3.41±0.2	3.42±0.13	3.35±0.19
IWTd (mm)	1.9±0.06	1.94±0.12	1.95±0.08	1.75±0.07
PWTs (mm)	3.51±0.11	3.59±0.15	3.47±0.1	3.49±0.1
PWTd (mm)	2.1±0.08	2.07±0.11	1.99±0.09	1.92±0.1
SWTs (mm)	3.37±0.1	3.4±0.13	3.5±0.03	3.49±0.08
SWTd (mm)	1.87±0.05	1.89±0.07	1.98±0.06	1.94±0.04

Values are presented as mean±S.E.M. (n=8-12). Sham: sham-operated group, CKD: chronic kidney disease group. **AWTd**: diastolic anterior wall thickness, **AWTs**: systolic anterior wall thickness **CO**: cardiac output, **E-velocity**: early ventricular filling velocity, **e'-velocity**: diastolic septal mitral annulus velocity, **EF**: ejection fraction, **HR**: heart rate, **IVCT**: isovolumic relaxation time, **IVRT**: isovolumic contraction time, **IWTd**: diastolic inferior wall thickness, **IWTs**: systolic inferior wall thickness, **LVEDV**: left ventricular end-diastolic volume, **LVESV**: left ventricular end-systolic volume, **PWTd**: diastolic posterior wall thickness, **PWTs**: systolic posterior wall thickness, **SV**: stroke volume, **SWTd**: diastolic septal wall thickness, **SWTs**: systolic septal wall thickness.

Supplementary Table S2 Body weight, tibia length, and organ weights at week 13

Parameter (unit)	Groups			
	Sham	CKD	CKD+losartan	CKD+mirabegron
Body weight (g)	454±10	466±10	454±13	459±18
Tibia length (cm)	4.2±0.03	4.25±0.03	4.22±0.04	4.26±0.04
Right ventricular weight (mg)	198±7	201±6	190±9	206±14
Heart weight (mg)	1161±40	1251±25	1118±20[#]	1335±84
Left ventricular weight (mg)	811±23	898±19*	774±14[#]	961±19*
Left kidney weight (mg)	1313±55	1647±90*	1492±62	1749±146*
Lung weight (mg)	1620±29	1693±37	1666±49	1828±90*

Values are presented as mean±S.E.M., *$p < 0.05$ vs. sham-operated group, [#]$p < 0.05$ vs. CKD group (n=7-10, One-Way ANOVA, Holm-Sidak *post hoc* test). Sham: sham-operated group, CKD: chronic kidney disease group.

Supplementary Figure S1

(a)

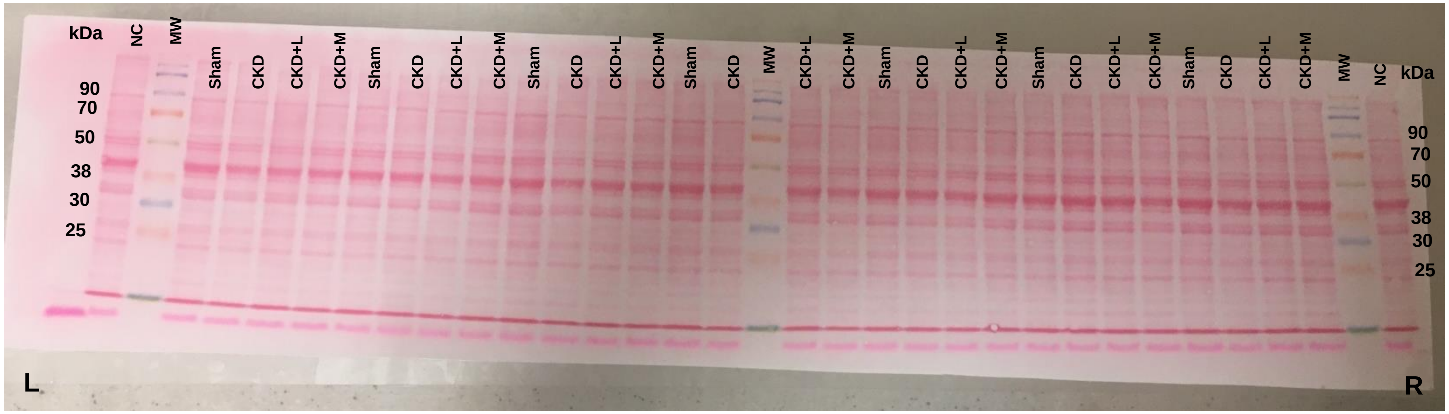

(b)

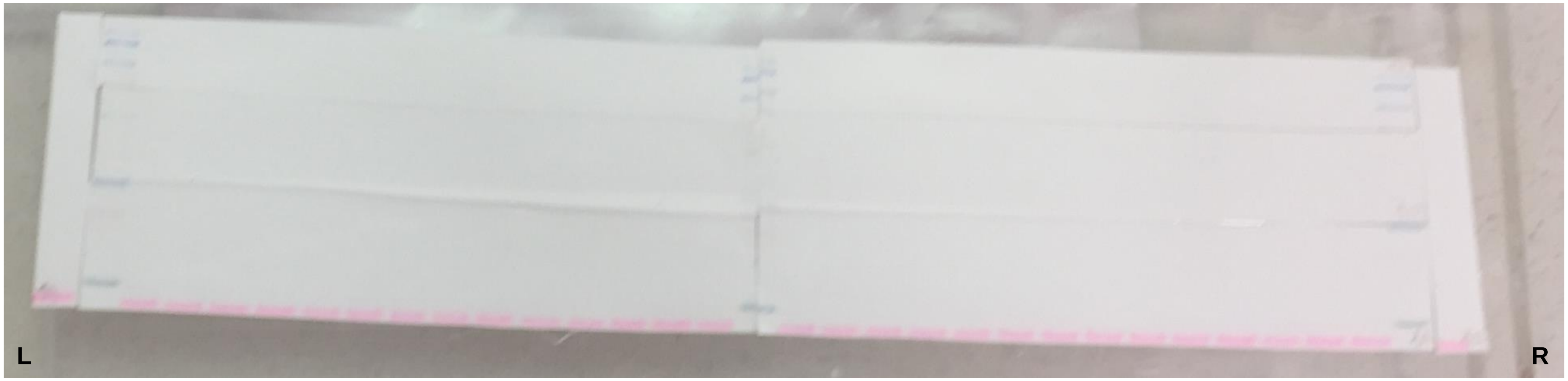

(a) Photo of the uncropped and unmodified Ponceau-stained membrane used later for the detection of beta3-adrenoceptor (β3-AR) and actin. The efficacy of the transfer of proteins onto a nitrocellulose membrane was checked using Ponceau staining.

(b) Photo of horizontally and vertically cut nitrocellulose membrane strips after incubation with primary (β3-AR and actin) and secondary (IRDye 800CW Goat Anti-Rabbit and IRDye 680RD Goat Anti-Mouse, respectively) antibodies, before scanning.

Sham: sham-operated group, CKD: chronic kidney disease group, CKD+L: losartan-treated chronic kidney disease group,
CKD+M: mirabegron-treated chronic kidney disease group, MW: molecular weight marker, NC: negative control, L: left, R: right.
(Images were captured by the camera of an Apple iPhone6.)

Supplementary Figure S2

Unmodified Western blot images of β3-AR and actin. Sham: sham-operated group, CKD: chronic kidney disease group, CKD+L: losartan-treated chronic kidney disease group, CKD+M: mirabegron-treated chronic kidney disease group, β3-AR: beta3-adrenoceptor, MW: molecular weight marker, NC: negative control, L: left, R: right. Scanned images were captured with the Odyssey CLx machine and exported with Image Studio 5.2.5 software. Different parts of the same membrane are divided by black lines. The membrane was physically cut in the middle of the molecular weight marker at lane 17 before scanning. Cropped images were used in Fig. 5a.

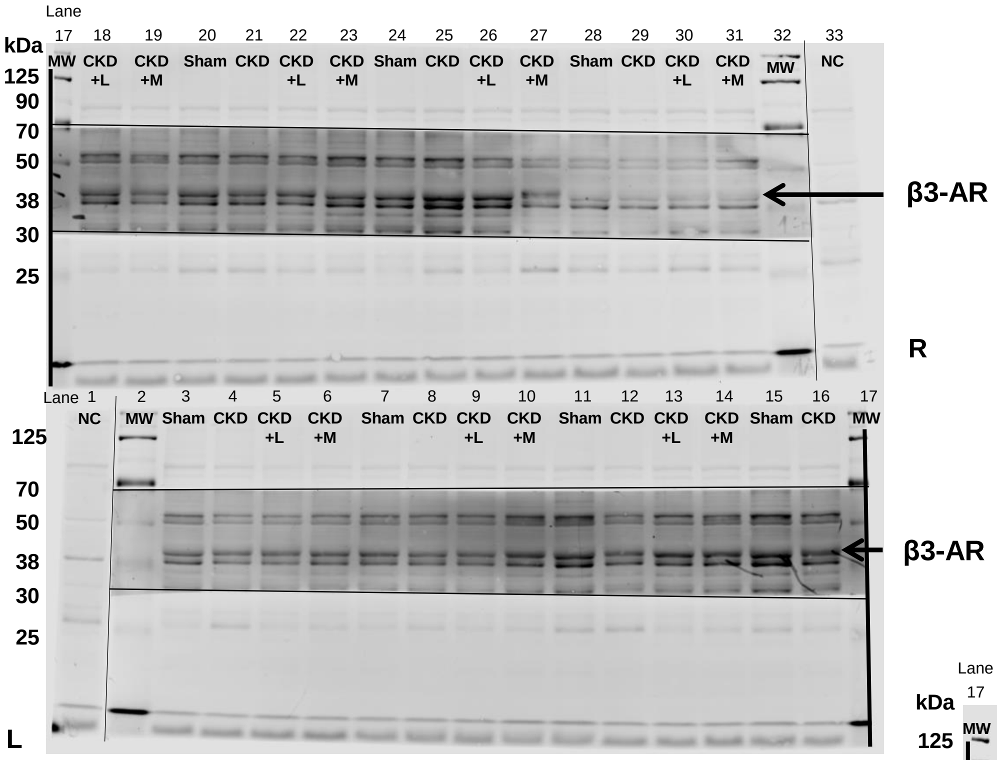

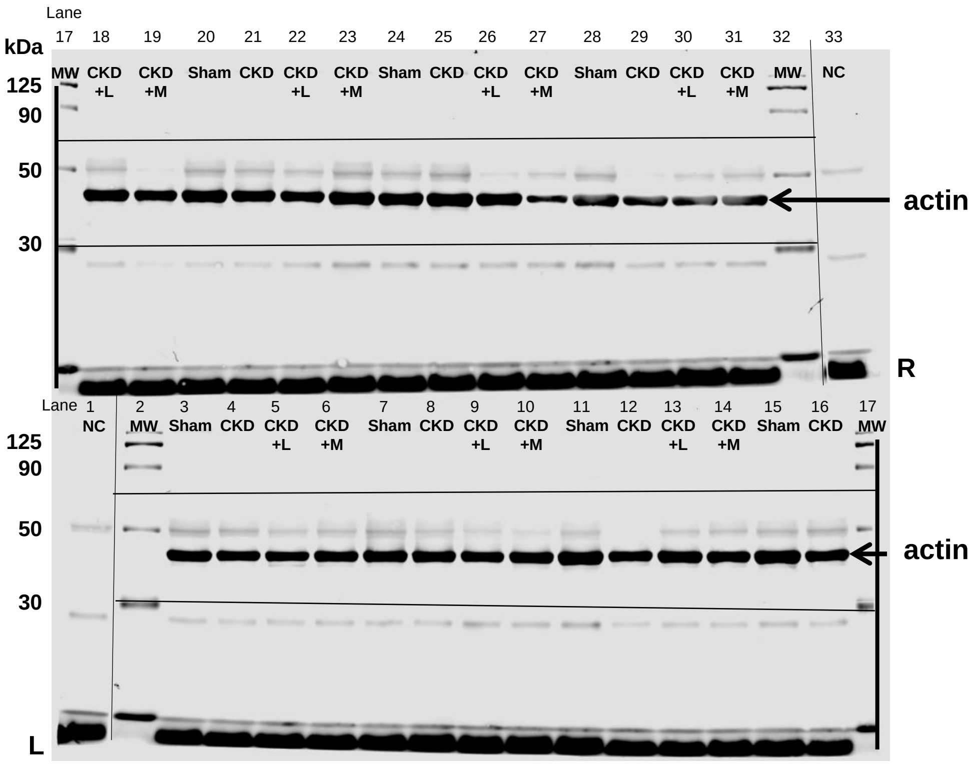

Supplementary Figure S3

(a)

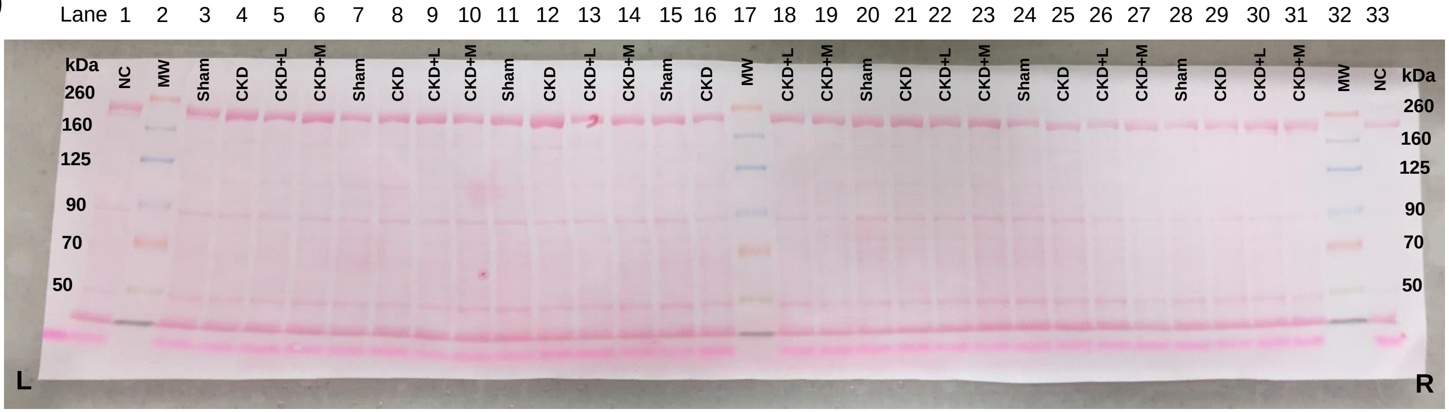

(b)

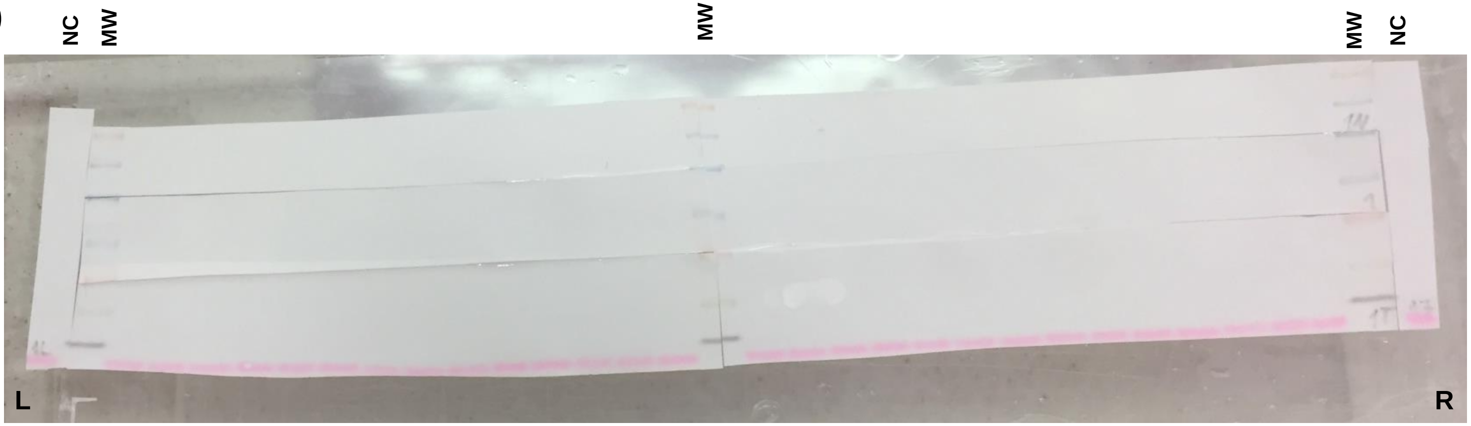

(a) Photo of the uncropped and unmodified Ponceau-stained membrane used later for the detection of endothelial nitric oxide synthase (eNOS) and tubulin. The efficacy of the transfer of proteins onto a nitrocellulose membrane was checked using Ponceau staining.

(b) Photo of horizontally and vertically cut nitrocellulose membrane strips after incubation with primary (eNOS and tubulin, respectively) and secondary (IRDye 800CW Goat Anti-Rabbit) antibodies, before scanning.

Sham: sham-operated group, CKD: chronic kidney disease group, CKD+L: losartan-treated chronic kidney disease group,
CKD+M: mirabegron-treated chronic kidney disease group, MW: molecular weight marker, NC: negative control, L: left, R: right.
(Images were captured by the camera of an Apple iPhone6.)

Supplementary Figure S4

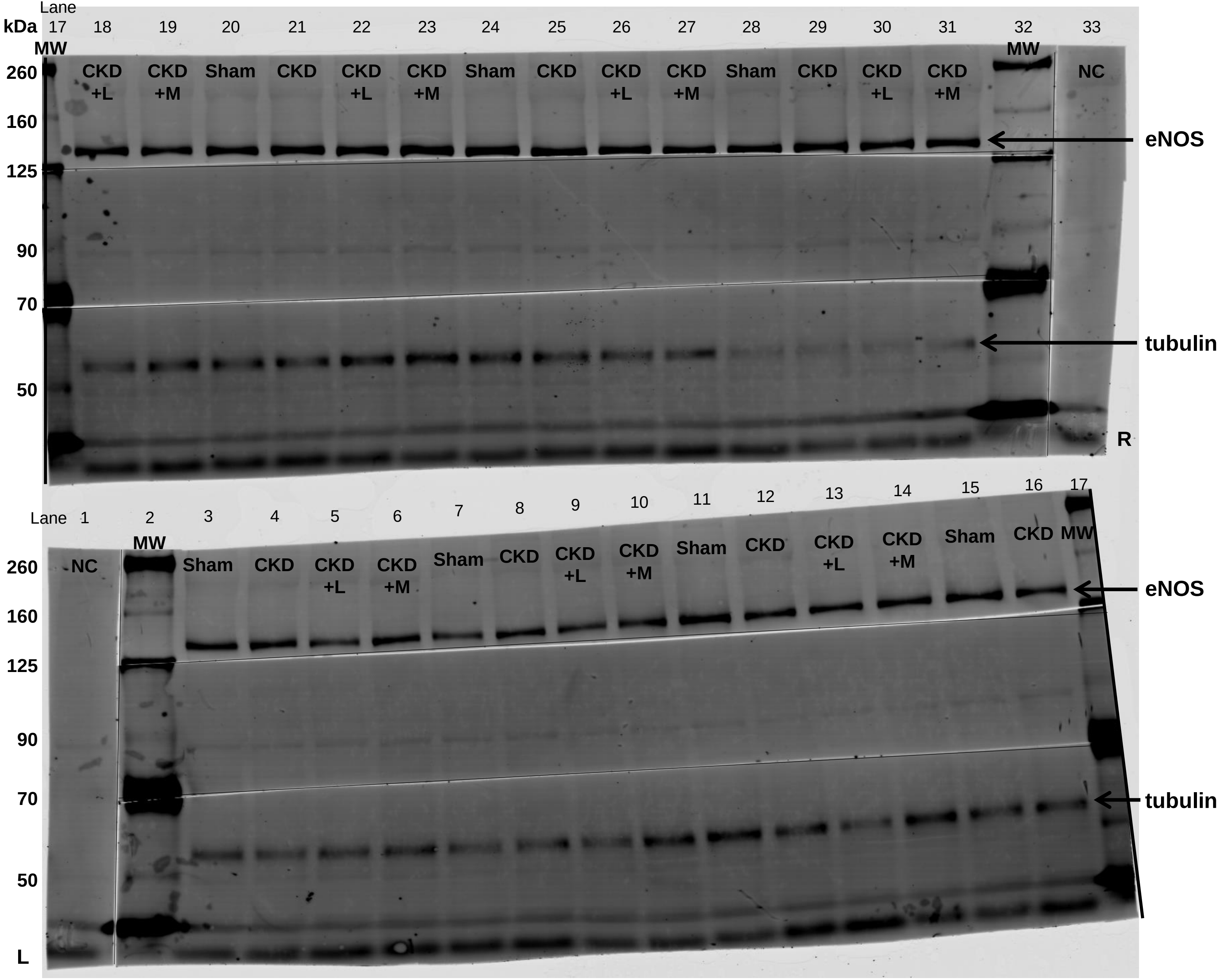

Unmodified Western blot images of eNOS and tubulin. Sham: sham-operated group, CKD: chronic kidney disease group, CKD+L: losartan-treated chronic kidney disease group, CKD+M: mirabegron-treated chronic kidney disease group, eNOS: endothelial nitric oxide synthase, MW: molecular weight marker, NC: negative control, L: left, R: right. Scanned images were captured with the Odyssey CLx machine and exported with Image Studio 5.2.5 software. Different parts of the same membrane are divided by black lines. The membrane was physically cut in the middle of the molecular weight marker at lane 17 before scanning. Cropped images were used in Fig. 5b and 5d.

Supplementary Figure S5

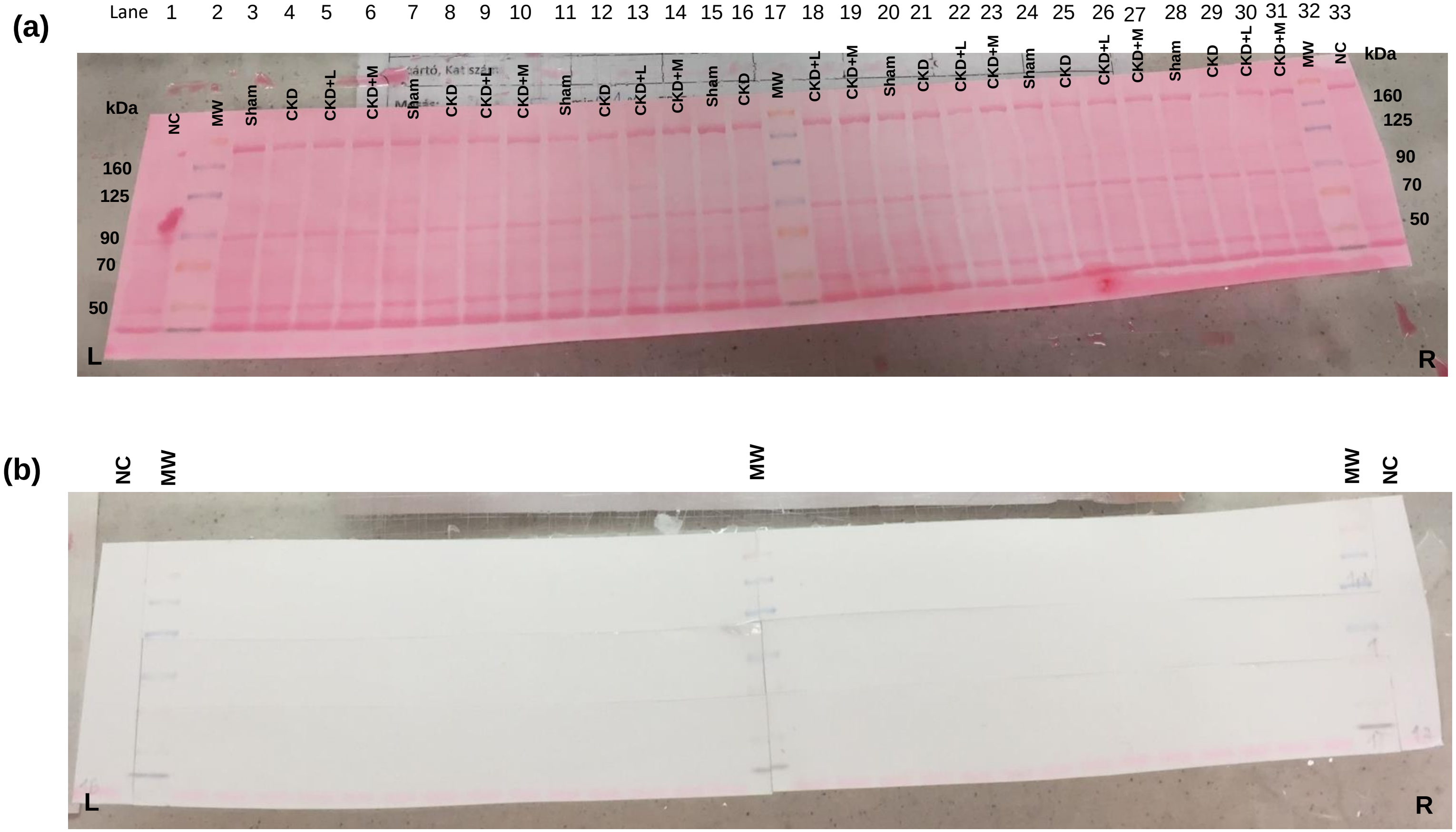

(a) Photo of the uncropped and unmodified Ponceau-stained membrane used later for the detection of phosphorylated endothelial nitric oxide synthase (p-eNOS) and tubulin. The efficacy of the transfer of proteins onto a nitrocellulose membrane was checked using Ponceau staining.

(b) Photo of horizontally and vertically cut nitrocellulose membrane strips after incubation with primary (p-eNOS and tubulin, respectively) and secondary (IRDye 800CW Goat Anti-Rabbit) antibodies, before scanning.

Sham: sham-operated group, CKD: chronic kidney disease group, CKD+L: losartan-treated chronic kidney disease group, CKD+M: mirabegron-treated chronic kidney disease group, MW: molecular weight marker, NC: negative control, L: left, R: right. (Images were captured by the camera of an Apple iPhone6.)

Supplementary Figure S6

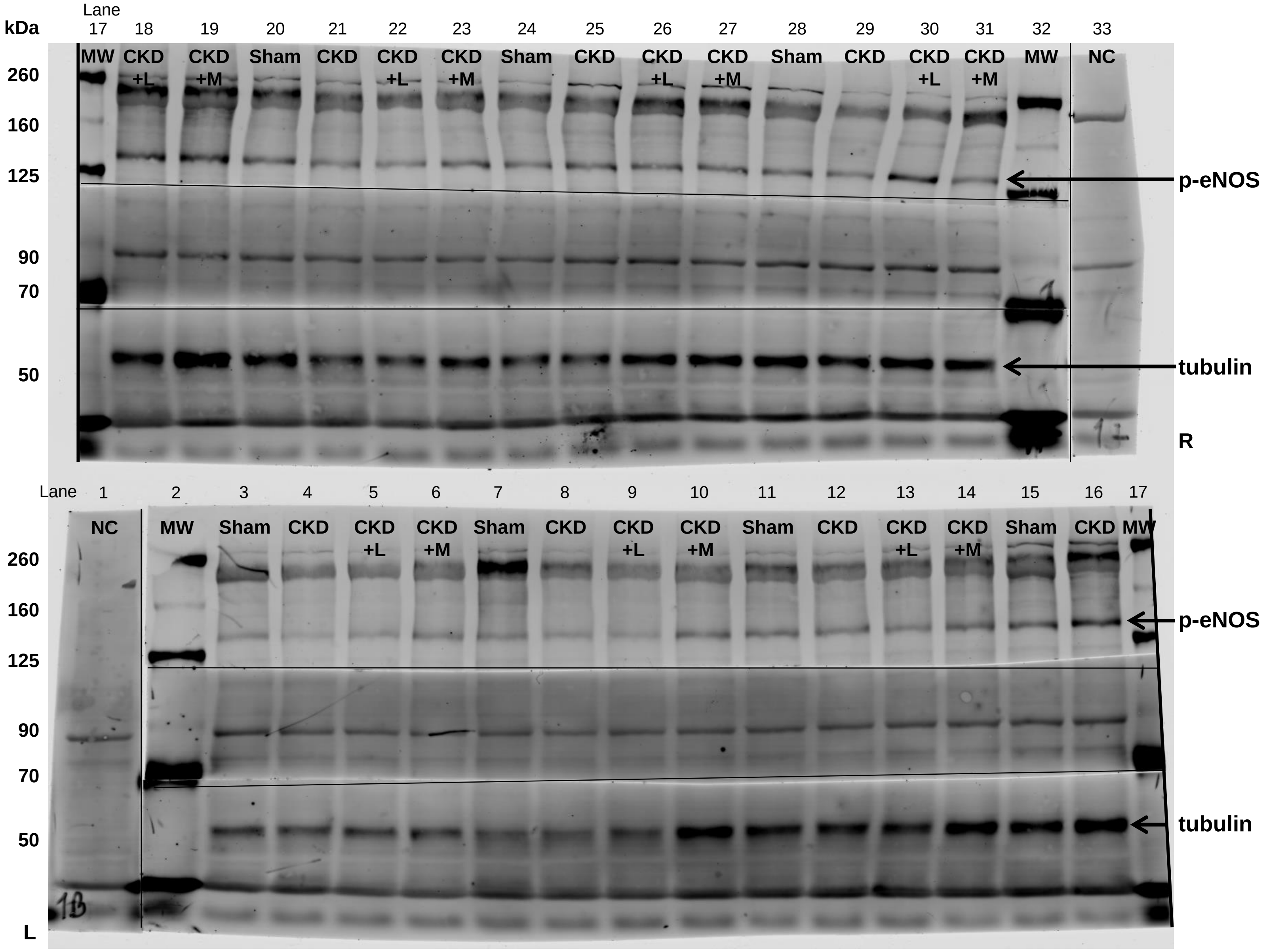

Unmodified Western blot images of p-eNOS and tubulin. Sham: sham-operated group, CKD: chronic kidney disease group, CKD+L: losartan-treated chronic kidney disease group, CKD+M: mirabegron-treated chronic kidney disease group, p-eNOS: phosphorylated endothelial nitric oxide synthase, MW: molecular weight marker, NC: negative control, L: left, R: right. Scanned images were captured with the Odyssey CLx machine and exported with Image Studio 5.2.5 software. Different parts of the same membrane are divided by black lines. The membrane was physically cut in the middle of the molecular weight marker at lane 17 before scanning. Cropped images were used in Fig. 5c and 5d.

(a)

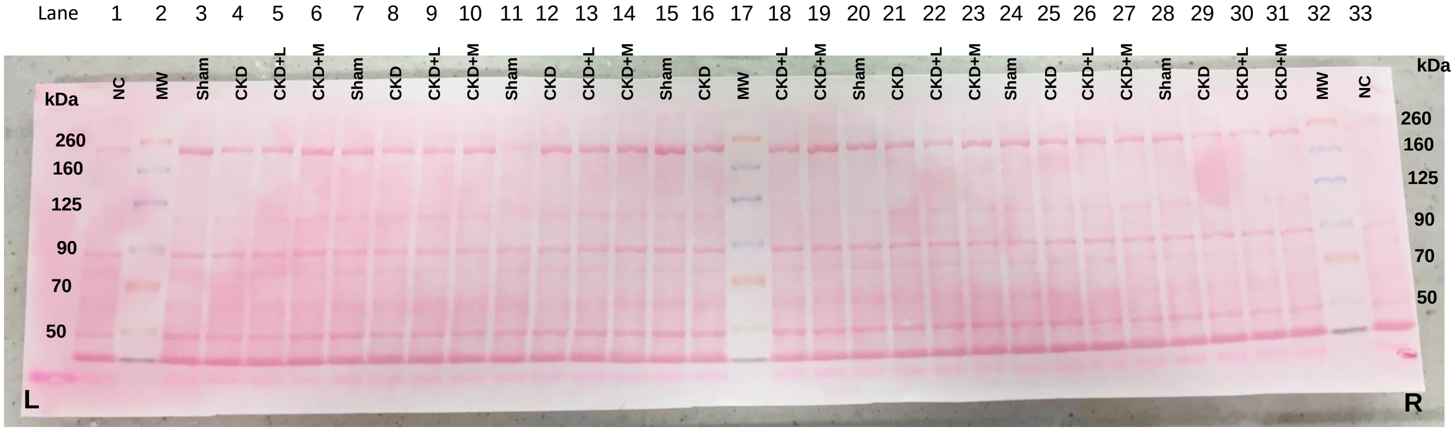

(b)

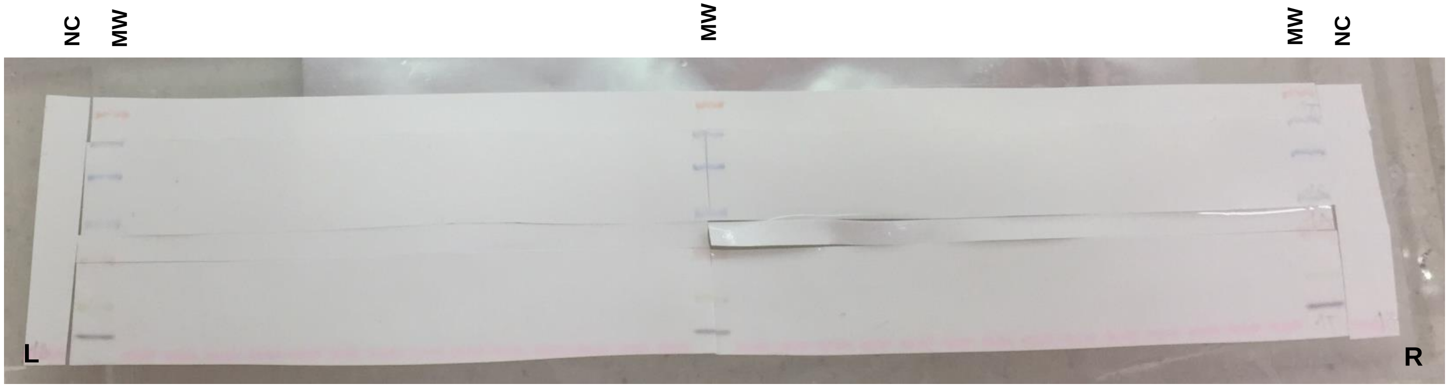

(a) Photo of the uncropped and unmodified Ponceau-stained membrane used later for the detection of sarcoplasmic reticulum ATPase (SERCA2a) and tubulin. The efficacy of the transfer of proteins onto a nitrocellulose membrane was checked using Ponceau staining.

(b) Photo of horizontally and vertically cut nitrocellulose membrane strips after incubation with primary (SERCA2a and tubulin, respectively) and secondary (IRDye 800CW Goat Anti-Rabbit) antibodies, before scanning.

Sham: sham-operated group, CKD: chronic kidney disease group, CKD+L: losartan-treated chronic kidney disease group,
CKD+M: mirabegron-treated chronic kidney disease group, MW: molecular weight marker, NC: negative control, L: left, R: right.
(Images were captured by the camera of an Apple iPhone6)

Supplementary Figure S8

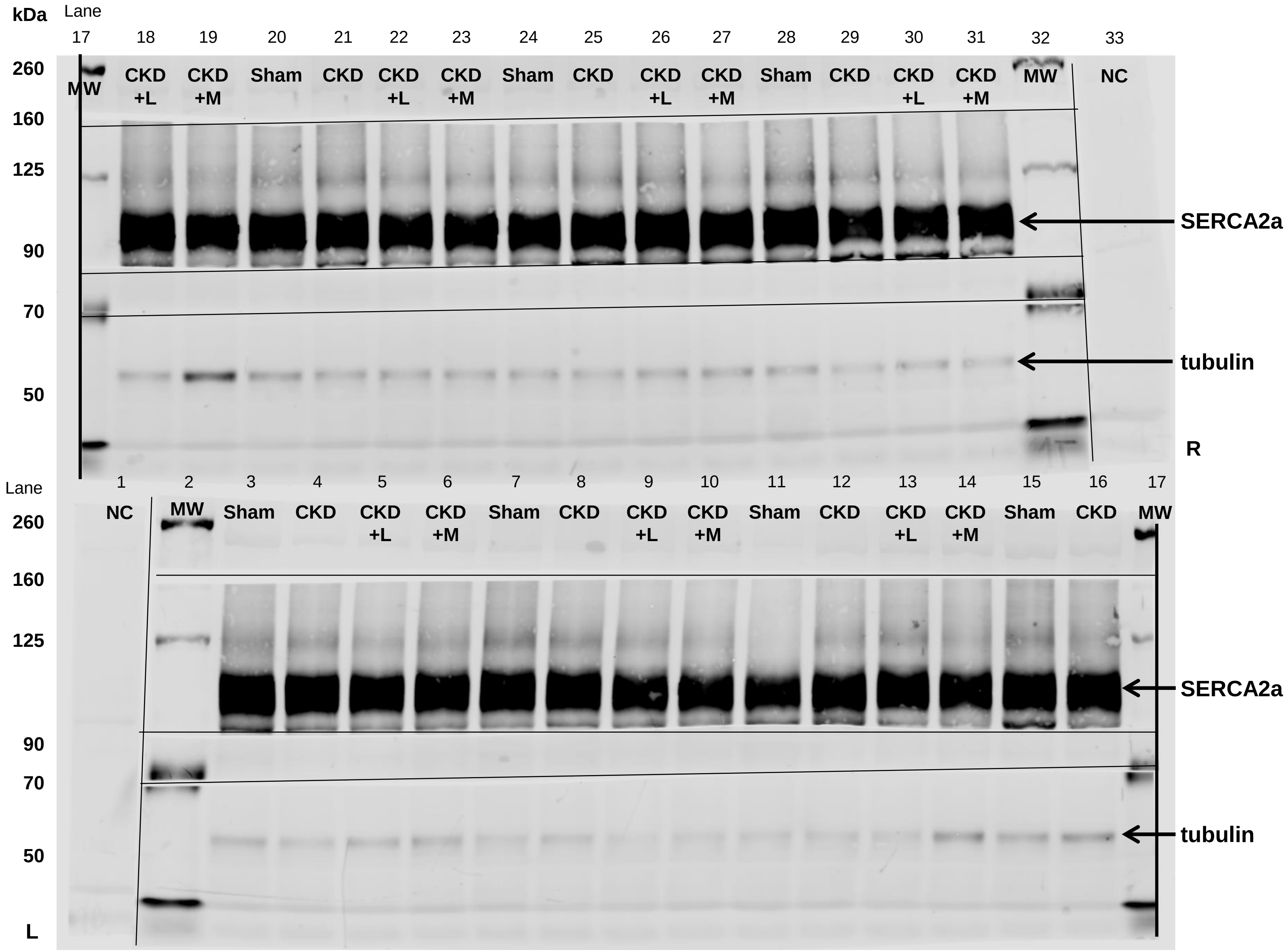

Unmodified Western blot images of SERCA2a and tubulin. Sham: sham-operated group, CKD: chronic kidney disease group, CKD+L: losartan-treated chronic kidney disease group, CKD+M: mirabegron-treated chronic kidney disease group, SERCA2a: sarcoplasmic reticulum ATPase, MW: molecular weight marker, NC: negative control, L: left, R: right. Scanned images were captured with the Odyssey CLx machine and exported with Image Studio 5.2.5 software. Different parts of the same membrane are divided by black lines. The membrane was physically cut in the middle of the molecular weight marker at lane 17 before scanning. Cropped images were used in Fig. 5e.

(a)

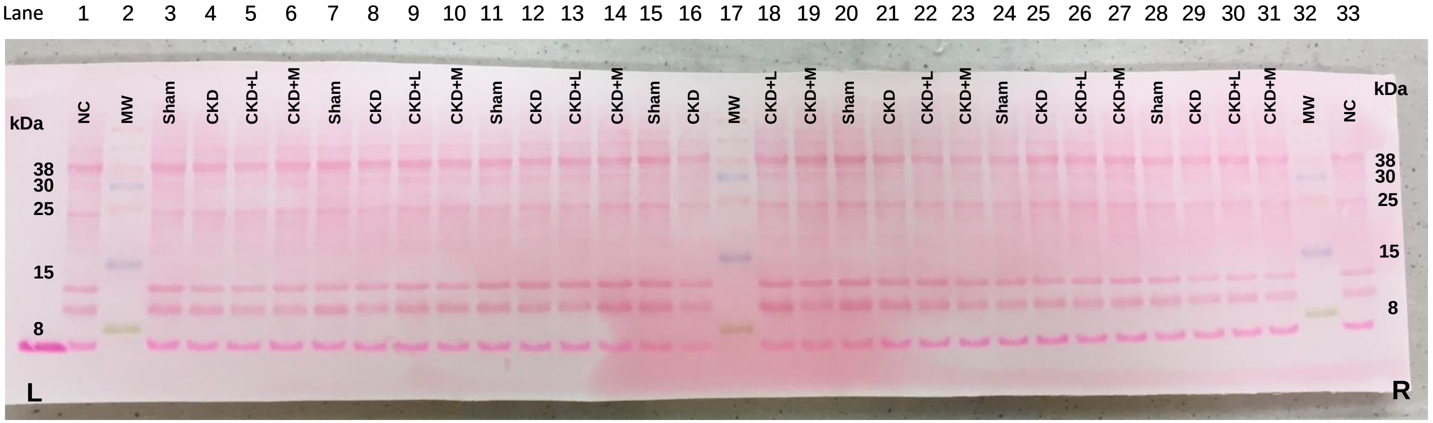

(b)

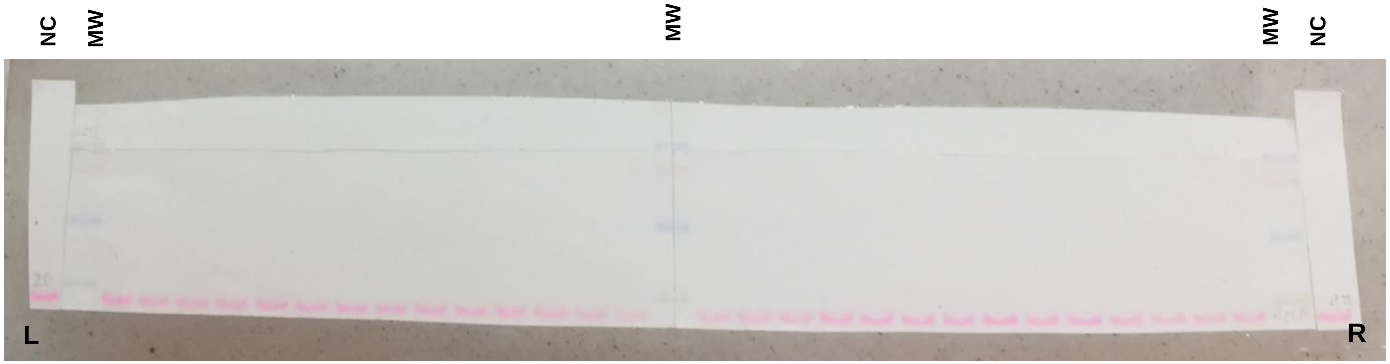

(a) Photo of the uncropped and unmodified Ponceau-stained membrane used later for the detection of phospholamban (PLN) and glyceraldehyde 3-phosphate dehydrogenase (GAPDH). The efficacy of the transfer of proteins onto a nitrocellulose membrane was checked using Ponceau staining.

(b) Photo of horizontally and vertically cut nitrocellulose membrane strips after incubation with primary (PLN and GAPDH, respectively) and secondary (IRDye 800CW Goat Anti-Rabbit) antibodies, before scanning.

Sham: sham-operated group, CKD: chronic kidney disease group, CKD+L: losartan-treated chronic kidney disease group, CKD+M: mirabegron-treated chronic kidney disease group, MW: molecular weight marker, NC: negative control, L: left, R: right. (Images were captured by the camera of an Apple iPhone6.)

Supplementary Figure S10

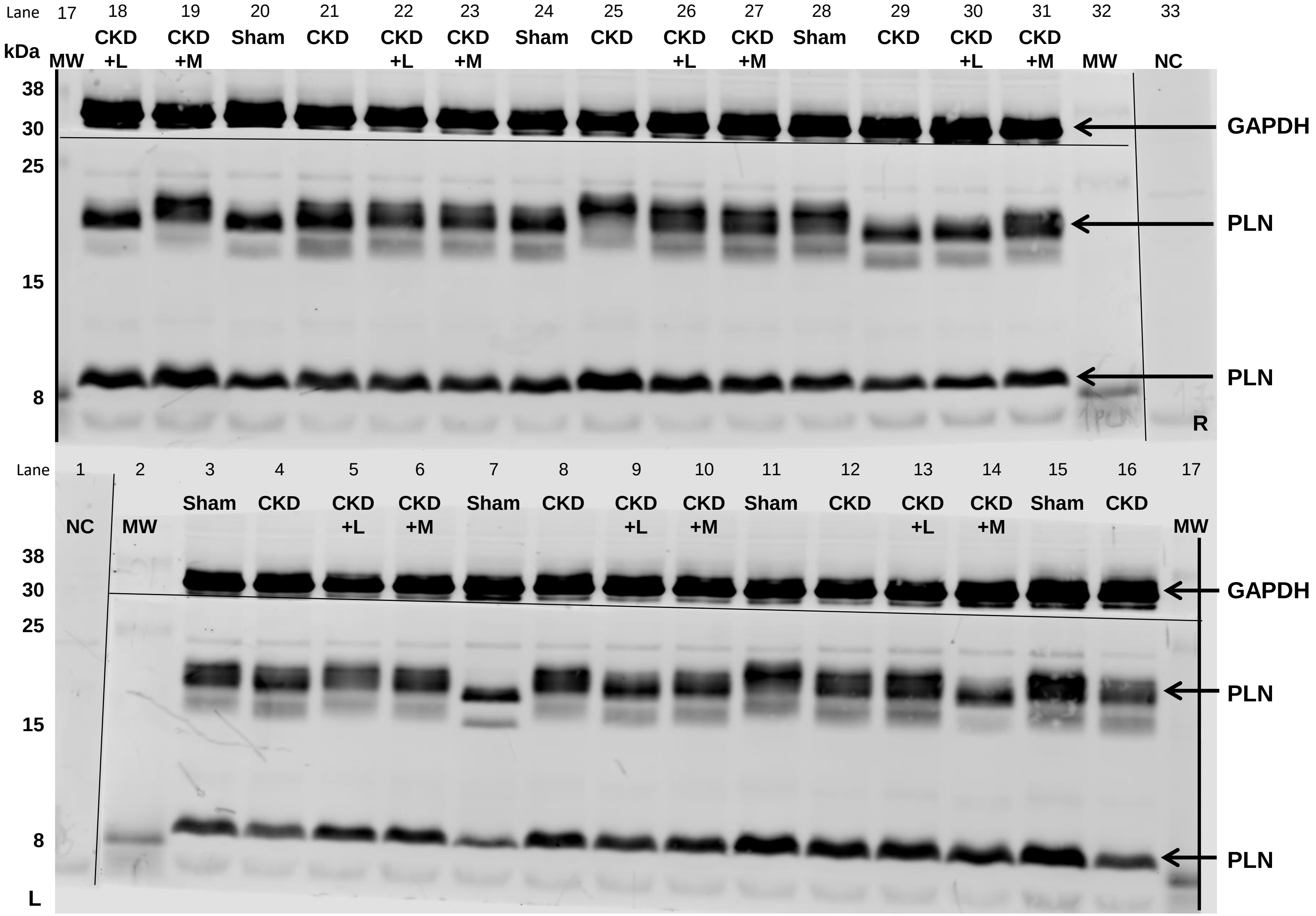

Unmodified Western blot images of PLN and GAPDH. Sham: sham-operated group, CKD: chronic kidney disease group, CKD+L: losartan-treated chronic kidney disease group, CKD+M: mirabegron-treated chronic kidney disease group, GAPDH: glyceraldehyde 3-phosphate dehydrogenase, PLN: phospholamban, MW: molecular weight marker, NC: negative control, L: left, R: right. Scanned images were captured with the Odysses CLx machine and exported with Image Studio 5.2.5 software. Different parts of the same membrane are divided by black lines. The membrane was physically cut in the middle of the molecular weight marker at lane 17 before scanning. Cropped images were used in Fig. 5f and 5h.

Supplementary Figure S11

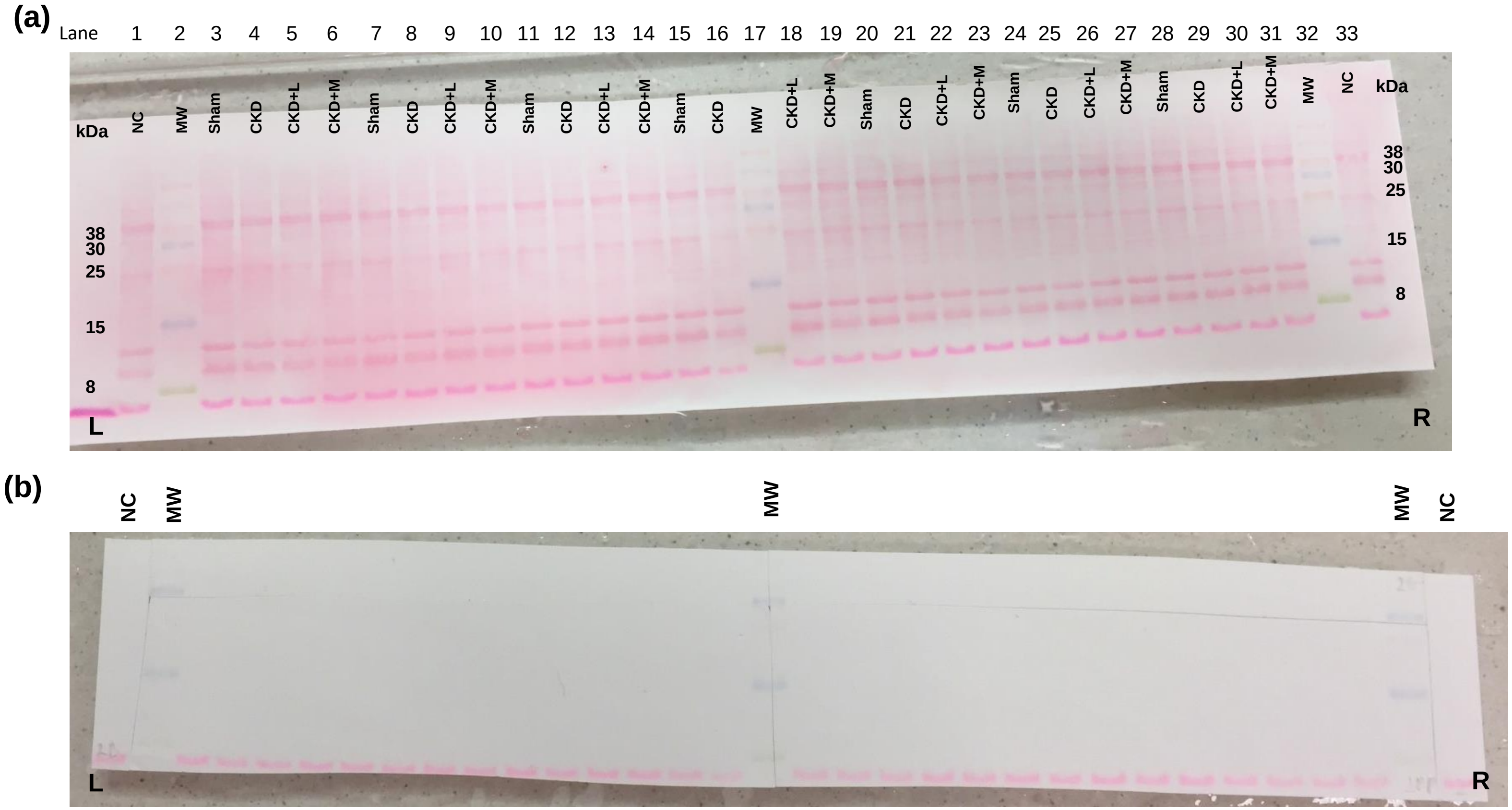

(a) The efficacy of the transfer of proteins onto a nitrocellulose membrane was checked using Ponceau staining. Photo of the uncropped and unmodified Ponceau-stained membrane used later for the detection of phosphorylated phospholamban (pPLN) and glyceraldehyde 3-phosphate dehydrogenase (GAPDH).

(b) Photo of horizontally and vertically cut nitrocellulose membrane strips after incubation with primary (pPLN and GAPDH, respectively) and secondary (IRDye 800CW Goat Anti-Rabbit) antibodies, before scanning.

Sham: sham-operated group, CKD: chronic kidney disease group, CKD+L: losartan-treated chronic kidney disease group, CKD+M: mirabegron-treated chronic kidney disease group, MW: molecular weight marker, NC: negative control, L: left, R: right. (Images were captured by the camera of an Apple iPhone6.)

Supplementary Figure S12

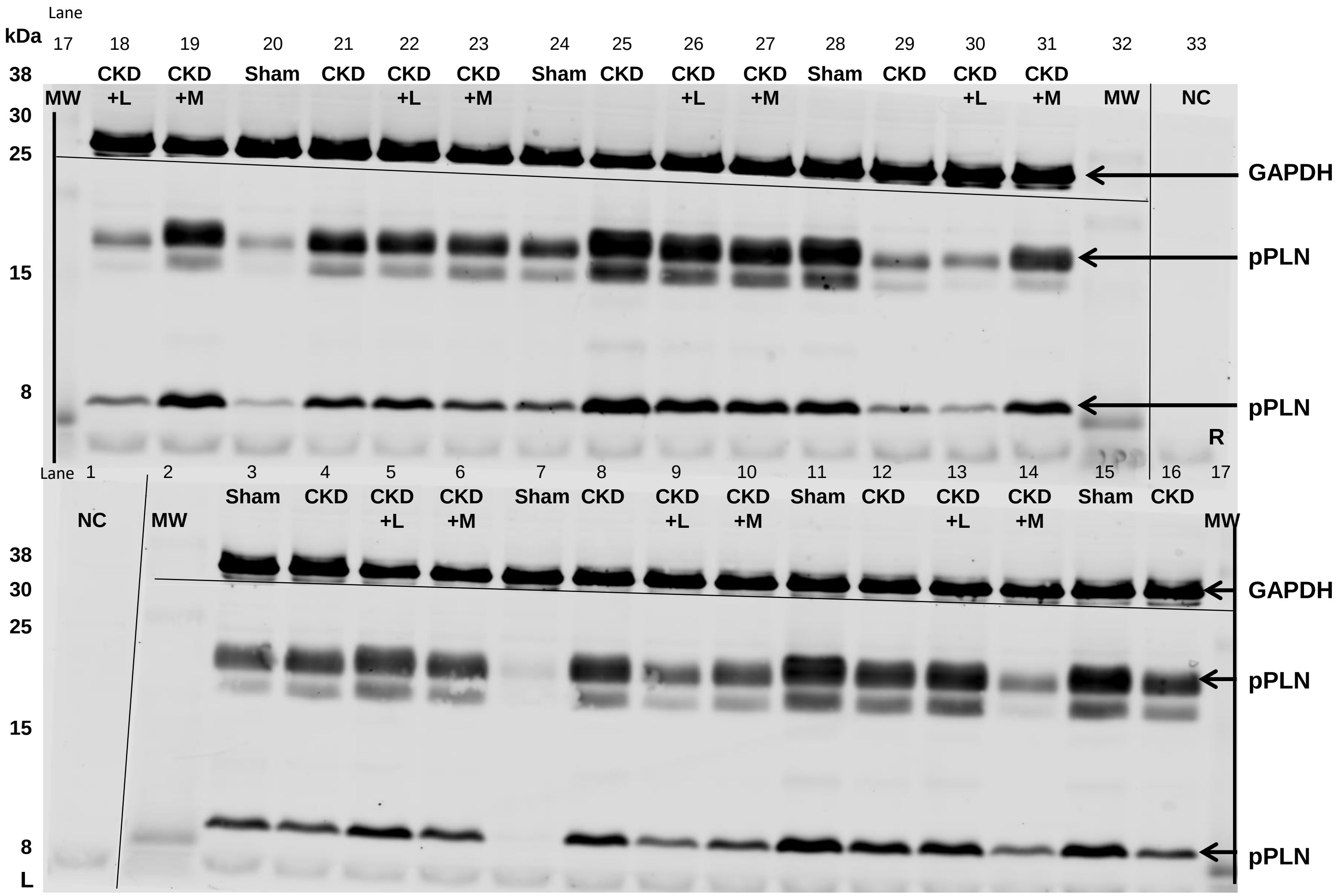

Unmodified Western blot images of pPLN and GAPDH. Sham: sham-operated group, CKD: chronic kidney disease group, CKD+L: losartan-treated chronic kidney disease group, CKD+M: mirabegron-treated chronic kidney disease group, GAPDH: glyceraldehyde 3-phosphate dehydrogenas, pPLN: phosphorylated phospholamban, MW: molecular weight marker, NC: negative control, L: left, R: right. Scanned images were captured with the Odyssey CLx machine and exported with Image Studio 5.2.5 software. Different parts of the same membrane are divided by black lines. The membrane was physically cut in the middle of the molecular weight marker at lane 17 before scanning. Cropped images were used in Fig. 5g and 5h.

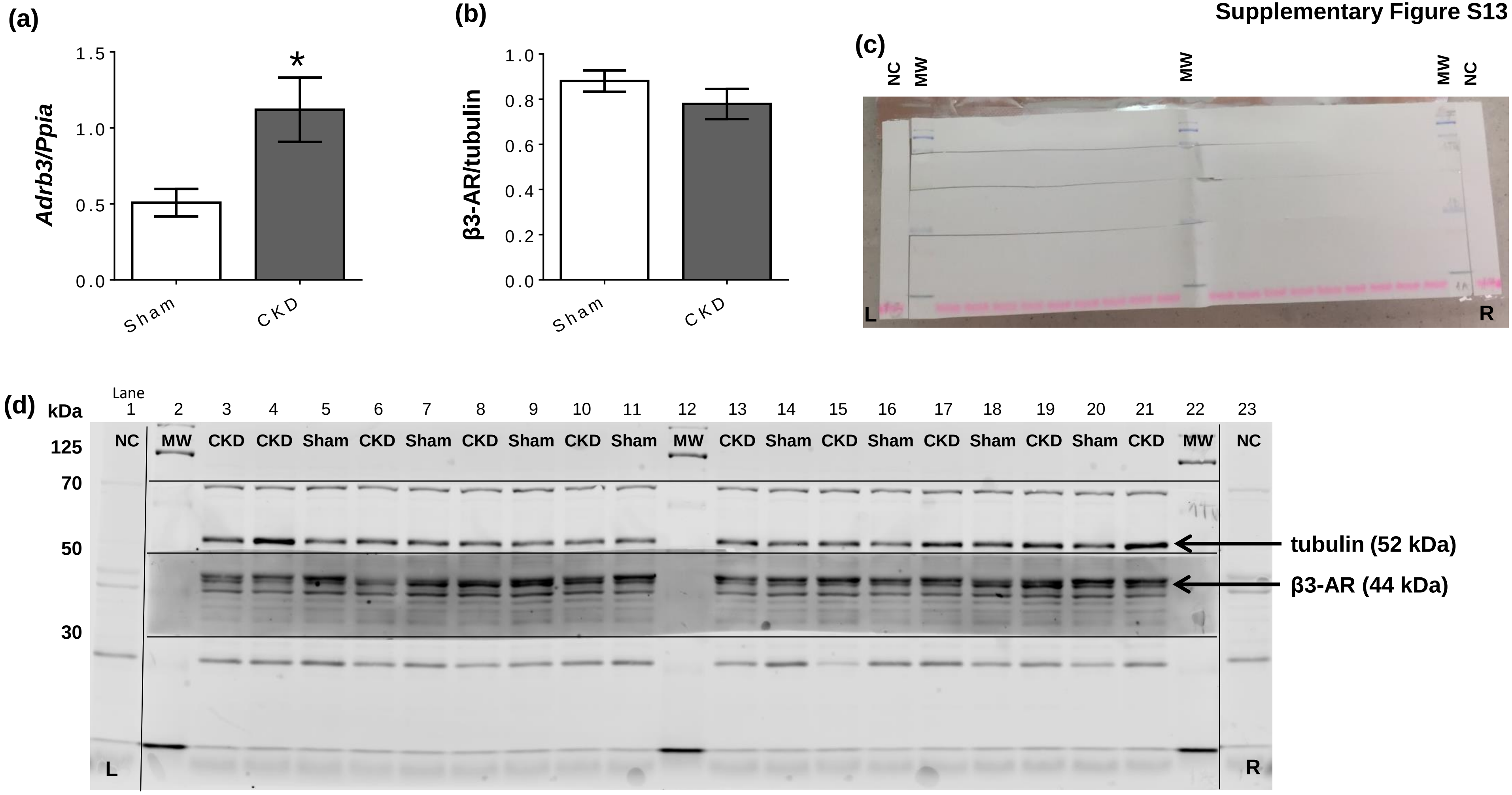

Left ventricular expression of β3-AR mRNA and protein in CKD 9 weeks after the 5/6th nephrectomy compared to the sham-operated group. (a) qRT-PCR results at week 9, **(b)** protein expression at week 9 assessed by Western blot, **(c)** photo of horizontally and vertically cut nitrocellulose membrane strips after incubation with primary (beta3-adrenoceptor (β3-AR) and tubulin, respectively) and secondary (IRDye 800CW Goat Anti-Rabbit) antibodies, before scanning, and **(d)** unmodified Western blot image. Different parts of the same membrane are divided by black lines.

Values are presented as mean±S.E.M., *p<0.05 (n=8-10, unpaired t-test). Sham: sham-operated group, CKD: chronic kidney disease group, MW: molecular weight marker, NC: negative control. The cut membrane was captured by the camera of an Apple iPhone6. The Western blot image was captured with the Odyssey CLx machine and exported with Image Studio 5.2.5 software.
